# Recent Advances in Transition Metal‐Based Metal‐Organic Frameworks for Hydrogen Production

**DOI:** 10.1002/smsc.202400446

**Published:** 2025-02-17

**Authors:** Ting Yang, Hua Zhang, Bo Pang, Jonathan W. C. Wong

**Affiliations:** ^1^ Research Centre for Eco‐Environmental Engineering, School of Environment and Civil Engineering Dongguan University of Technology Dongguan 523830 China; ^2^ School of Chemical Engineering Jiangxi Normal University Nanchang 330022 China; ^3^ Department of Food Science and Technology National University of Singapore Singapore 117542 Singapore; ^4^ Department of Materials and Environmental Chemistry Stockholm University 10691 Stockholm Sweden

**Keywords:** electrocatalysis, hydrogen production, photocatalysis, renewable energies, transition metal‐based metal‐organic frameworks

## Abstract

The escalating global energy demand and the imperative to mitigate carbon emissions have intensified the pursuit for sustainable energy solutions, with hydrogen emerging as a pivotal clean energy carrier. Transition metal‐based metal‐organic frameworks (MOFs) have garnered significant attention for their potential in efficient hydrogen production due to their high surface area, tunable porosity, and versatile catalytic properties. Despite notable advancements in MOF synthesis, critical challenges related to stability, electrical conductivity, and scalability continue to hinder their widespread application. This review provides a comprehensive analysis of recent progress in the design and synthesis of transition metal‐based MOFs, emphasizing their role in electrocatalytic and photocatalytic hydrogen production. Key synthetic strategies and their influence on catalytic performance are systematically discussed, alongside the identification of existing limitations and knowledge gaps. By highlighting these critical areas and proposing pathways for future research, this review aims to accelerate the practical integration of MOFs into the emerging hydrogen economy.

## Graphical abstract

An organized outline for a comprehensive document that explores the synthesis, properties, and applications of metal‐organic frameworks, focusing on metal doping, organic linkers, ligand functionalization, and their roles in photocatalysis and electrocatalysis, is provided.

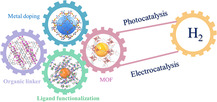

## 1 Introduction

The rapid growth of the global population and the acceleration of industrialization have led to a continuous increase in energy demand. Simultaneously, over‐reliance on traditional fossil fuels has triggered severe energy crises and environmental pollution problems, necessitating the exploration of sustainable energy solutions.^[^ ^1^ ^]^ Hydrogen production via water splitting has emerged as a promising clean and renewable energy conversion technology, contributing to the reduction of greenhouse gas emissions and the mitigation of global climate change.^[^ ^2^ ^]^ As a clean energy carrier, hydrogen boasts a high energy density and extensive application potential, including fuel cells, chemical synthesis, and transportation.^[^ ^3^ ^]^ The efficient production of hydrogen through water splitting represents a critical step in realizing the hydrogen economy and advancing hydrogen energy technology.^[^ ^4^ ^]^

Water splitting consists of two key half reactions: the hydrogen evolution reaction (HER) and oxygen evolution reaction (OER).^[^ ^5^ ^]^ These two reactions proceed under electrochemical conditions and can be driven by renewable energy sources such as solar and wind, converting water into hydrogen and oxygen efficiently.^[^ ^6^ ^]^ Employing efficient catalysts is critical to enhancing reaction rates and reducing energy consumption in water splitting processes.^[^ ^7^ ^]^ However, the current reliance on precious metal catalysts such as platinum for HER and iridium for OER faces challenges related to high costs and limited availability.^[^ ^8^ ^]^ Consequently, the development of low‐cost, efficient, and durable transition metal (TM)‐based catalysts is vital for the commercial viability of water‐splitting technology.^[^ ^9^ ^]^

TM catalysts have gained significant attention due to their rich elemental diversity, tunable electronic structures, and relatively low cost.^[^ ^10^ ^]^ In particular, TMs such as iron (Fe), cobalt (Co), and nickel (Ni) exhibit catalytic properties comparable to those of precious metals, while being more abundant and cost‐effective.^[^ ^11^ ^]^ TM‐based metal‐organic frameworks (MOFs) have emerged as a promising class of materials for catalytic hydrogen production, attributed to their unique structural and chemical properties.^[^ ^12^ ^]^ These materials exhibit distinct advantages for hydrogen production, including their high surface area, tunable porosity, and versatile catalytic properties. The intrinsic properties of TM, such as variable oxidation states, unfilled *d‐*orbitals, and the capacity to form multinuclear metal clusters, enhance the catalytic efficiency and stability of MOFs.^[^ ^13^ ^]^ These properties position TM‐based MOFs as advantageous in applications requiring catalysis, adsorption, and electronic conduction.^[^ ^14^ ^]^ First, the multiple oxidation states of TMs provide MOFs with the ability to adjust electronic properties and coordination environments, tailoring them to meet the specific demands of diverse catalytic reactions.^[^ ^15^ ^]^ For example, cobalt‐based MOFs, such as Co‐MOF‐74, leverage the variable valence of Co to achieve efficient HER performance at low overpotentials in electrocatalytic water splitting.^[^ ^16^ ^]^ Similarly, the unfilled *d‐*orbitals of TMs serve as abundant active sites and facilitate electron‐accepting and ‐donating processes essential for intermediate stabilization and charge transfer.^[^ ^17^ ^]^ For example, Fe‐MOF‐74 utilizes the *d*‐orbitals of iron to provide highly effective catalytic sites for HER, demonstrating the pivotal role of TM electronic configurations in catalysis.^[^ ^18^ ^]^ Furthermore, the ability of TMs to form multinuclear metal clusters enhances the structural stability of MOFs, enabling them to maintain their integrity under harsh catalytic conditions.^[^ ^14^ ^]^ UiO‐66‐type MOFs, which incorporate Zr_6_O_8_ clusters, exemplify this stability, exhibiting exceptional thermal and chemical resilience due to the high connectivity and robustness of their multinuclear clusters.^[^ ^19^ ^]^ This structural advantage is particularly critical for the long‐term operation of catalytic systems. In addition, the pore structure of TM‐based MOFs can be precisely engineered by selecting specific metal centers and organic ligands to achieve selective adsorption and separation based on molecular size and shape.^[^ ^20^ ^]^ For instance, ZIF‐8 utilizes its tailored pore size to achieve efficient gas molecule adsorption, showcasing the versatility of MOFs in selective adsorption applications.^[^ ^21^ ^]^

The multifunctionality of TM‐based MOFs further enhances their utility. These materials often combine catalytic activity with other functional properties, such as gas storage and electrical conductivity, broadening their application potential.^[^ ^22^ ^]^ For example, Ni_3_(HITP)_2_ integrates gas storage capacity with high electrocatalytic efficiency due to its intrinsic electrical conductivity. This multifunctional nature makes MOFs particularly suitable for integrated energy systems and hybrid applications.^[^ ^23^ ^]^ The abundant and cost‐effective nature of TMs, such as Fe, Co, and Ni, also contributes to the economic feasibility of MOFs, providing a sustainable alternative to precious metal catalysts.^[^ ^24^ ^]^ In terms of environmental and economic sustainability, TM‐based MOFs offer significant advantages. Their recyclability and stability reduce maintenance costs and align with the principles of environmental friendliness.^[^ ^25^ ^]^ Furthermore, advancements in chemical synthesis and characterization techniques have enabled researchers to fine tune the design and optimize the performance of MOFs, offering valuable insights into catalytic mechanisms and guiding the development of next‐generation catalysts.^[^ ^26^ ^]^

Despite ongoing challenges in optimizing stability, conductivity, and scalability, TM‐based MOFs exhibit broad application prospects for hydrogen production. Their unique combination of structural, electronic, and functional properties positions them as a promising material class for advancing hydrogen production technologies.^[^ ^27^ ^]^ This article aims to provide a comprehensive review of the latest research progress in this field, analyzing the potential and challenges associated with MOF‐based hydrogen production technologies and offering insights into future research directions. Through an in‐depth examination of existing literature, this review highlights the critical role of MOFs in hydrogen production and their potential contribution to global energy transformation and sustainability efforts.

## 2 Overview and Classification of TM‐Based MOFs

**Figure 1:**
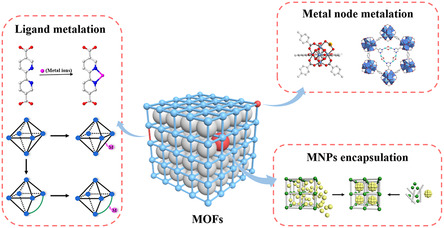
Strategies developed for the metalation of MOFs. Reproduced with permission.^[^ ^29^ ^]^ Copyright 2024, Royal Society of Chemistry.

As a class of porous crystalline materials, TM‐based MPFs are formed by TM‐based ions or clusters connected to organic ligands via coordination bonds (**Figure** **1**).^[^ ^28^, ^29^ ^]^ They have high chemical and structural tunability, excellent thermal stability, ultrahigh porosity, and large specific surface area, making them ideal for a range of applications, including hydrogen production.^[^ ^30^ ^]^ The TM‐based MOFs can be classified based on their constituent elements, structural features, pore properties, or functional attributes, providing flexibility in their design and synthesis.^[^ ^31^ ^]^

### 2.1 Classification Criteria of TM‐Based MOFs

MOFs can be categorized by their metal nodes, organic ligands, or structural motifs.^[^ ^32^ ^]^ For example, metal centers such as aluminum, iron, and copper influence MOF stability, catalytic activity, and chemical properties.^[^ ^33^ ^]^ Ligands, which may be monofunctional or multidentate, dictate the coordination geometry and pore characteristics of the framework.^[^ ^34^ ^]^ Additionally, classification based on pore control (e.g., size, shape, porosity, and active sites) or functional diversity, such as multivariate MOFs containing various metal ions or ligands, enables customization for specific applications.^[^ ^35^ ^]^ Structural motifs like rigid secondary building units or defect‐engineered frameworks enhance stability, porosity, and catalytic efficiency.^[^ ^36^ ^]^ For example, Zr‐based MOFs (e.g., UiO‐66) exhibit superior thermal and chemical stability, while Cu‐based MOFs (e.g., HKUST‐1) with exposed metal centers and Fe‐based MOFs (e.g., MIL‐53(Fe)) with flexible frameworks are particularly effective for catalytic hydrogen production.^[^ ^37^ ^]^

### 2.2 Ligands in TM‐Based MOFs

Organic ligands play a pivotal role in defining the structural and functional characteristics of MOFs. Based on the numbers and types of coordination sites, ligands are classified into monofunctional ligands and multidentate ligands.^[^ ^38^ ^]^ Both types offer unique advantages for tailoring the properties and performance of MOFs.

#### 2.2.1 Monofunctional Ligands

Monofunctional ligands contain a single functional group that coordinates with the metal center, typically through atoms with lone pairs of electrons, such as oxygen, nitrogen, or sulfur.^[^ ^39^ ^]^ These ligands are characterized by several defining features.^[^ ^40^ ^]^

##### Single‐Coordination Site

Monofunctional ligands possess only one functional group that can directly coordinate with the metal center.

##### Simple Structure

Compared to multidentate ligands, monofunctional ligands have a more straightforward structure, usually involving a single‐coordination site.

##### Functional Specificity

Despite their simplicity, monofunctional ligands play a crucial role in constructing MOF structures by determining the primary connection mode and the coordination environment around the metal center.

##### Easy of Synthesis

These ligands are typically easy to synthesize and widely available, making them popular for MOF synthesis.

##### Tunable Properties

By selecting different monofunctional ligands, the pore size, shape, and properties of MOFs can be adjusted, influencing their performance in applications such as gas adsorption, separation, and catalysis.

These characteristics make monofunctional ligands indispensable in the design and synthesis of MOFs, enabling precise control over their properties through careful selection and design.

#### 2.2.2 Multidentate Ligands

Multidentate ligand MOF materials refer to porous organic–inorganic hybrid materials formed by self‐assembly of organic ligands with multiple coordination sites and metal ions or clusters.^[^ ^41^ ^]^ These ligands can coordinate with multiple metal centers due to their numerous functional groups, resulting in more complex and stable MOF structures.^[^ ^42^ ^]^ Functionalized multidentate ligands have significantly enhanced the diversity of MOF structures and performance.^[^ ^43^ ^]^ These ligands not only enrich the chemical functionality of TM‐based MOFs by introducing functional groups such as phenolic, aldehyde/ketone, or urea/thiourea, but also improve pore properties and chemical stability by forming hydrogen bonds, participating in dynamic covalent chemistry, and enhancing adsorption performance.^[^ ^44^ ^]^ These improvements are particularly crucial for hydrogen adsorption, separation, and storage applications.

The incorporation of polynuclear ligands, which form stable coordination bonds with multiple metal centers, further increases pore complexity and material stability.^[^ ^43^ ^]^ The synergistic effects between metal centers improve the overall performance of MOFs, particularly in electrocatalytic hydrogen production.^[^ ^45^ ^]^ For example, in UiO‐66, the phenolic hydroxyl groups of the ligand form stable coordination bonds with zirconium ions, while noncoordinating hydroxyl groups act as hydrogen bond donors or acceptors, enhancing the framework's structural rigidity and providing additional sites for gas molecule adsorption.^[^ ^46^ ^]^ This dual functionality is particularly beneficial for hydrogen adsorption and storage, highlighting its significance in hydrogen energy storage and transportation.^[^ ^47^ ^]^

In addition, multifunctional ligands containing porphyrin or phthalocyanine derivatives demonstrate great potential in photocatalytic hydrogen production.^[^ ^48^ ^]^ These ligands serve as photosensitizers, absorbing light energy and exciting electrons, while their chemical diversity allows for adjustments in the light absorption range and electron transfer capabilities.^[^ ^49^ ^]^ This tunability enables the optimization of MOFs for specific photocatalytic reactions, thereby improving the efficiency of hydrogen production. Overall, the application of functionalized and multidentate ligands in MOFs introduces greater flexibility and diversity in material design and offers new strategies for addressing key scientific challenges in energy storage, catalysis, and separation.

### 2.3 Representative MOF Ligands

#### 2.3.1 Carboxylate‐Based Ligands

**Figure 2:**
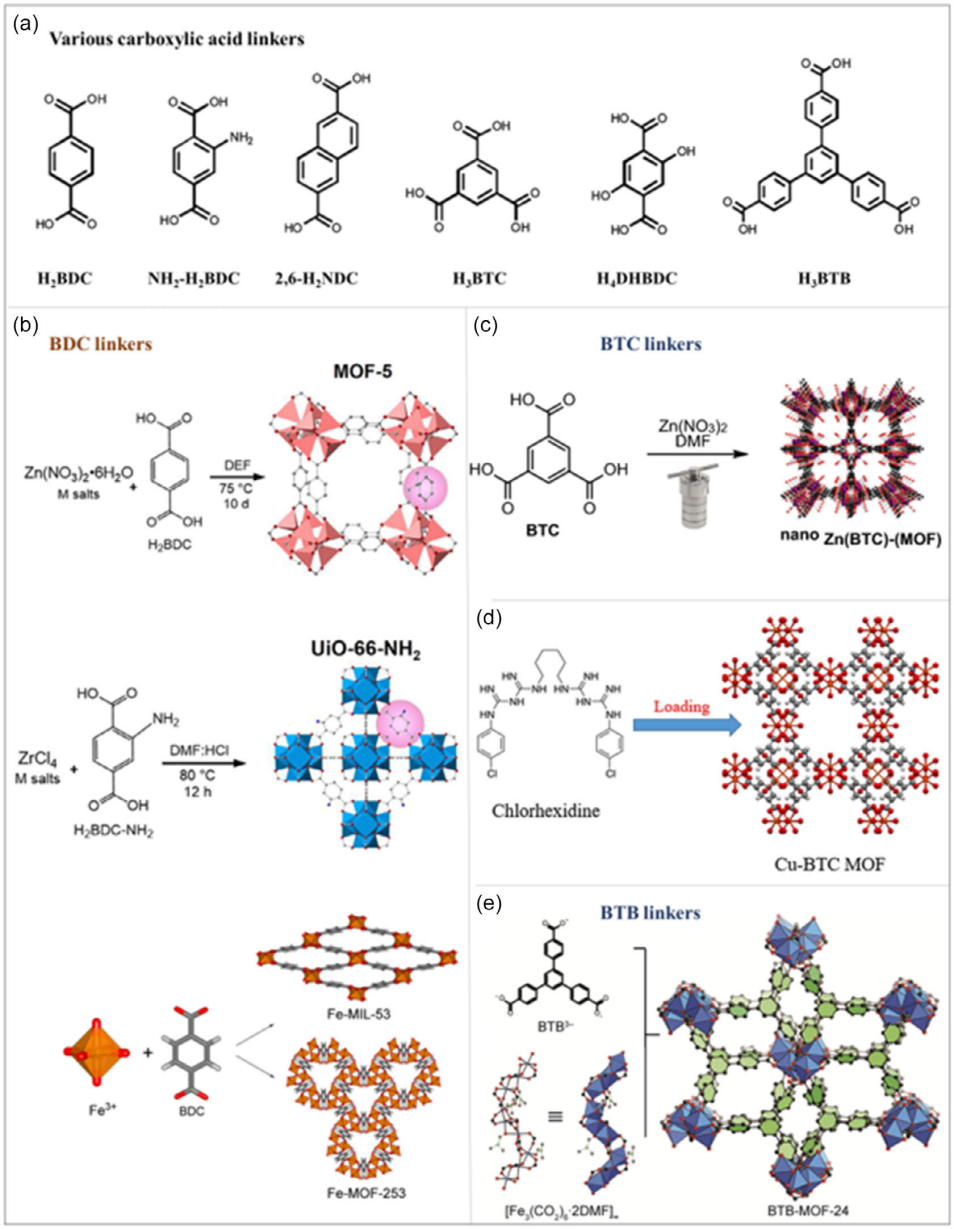
a) Structures of various carboxylic acid linkers for MOFs and the schematic reaction for synthesizing MOFs with carboxylic acid ligands. b) BDC ligands. Reproduced with permission.^[^ ^132^ ^]^ Copyright 2022, Elsevier B.V.; Copyright 2023, Elsevier B.V. c,d) BTC ligands. Reproduced with permission.^[^ ^133^ ^]^ Copyright 2023, Springer Nature; Copyright 2022, Springer Nature. e) BTB linkers. Reproduced with permission.^[^ ^134^ ^]^ Copyright 2022, Elsevier Inc.

Carboxylate‐based ligands in MOFs refer to organic molecules containing carboxylate functional groups (—COOH), which form coordination bonds with metal ions and play a central role in constructing MOF frameworks.^[^ ^50^ ^]^ These ligands are generally categorized into two main types: simple carboxylic acids and polycarboxylic acids, each contributing distinct characteristics and functionalities to MOFs (**Figure** **2**).

Simple carboxylic acids, such as acetic acid (CH_3_COOH) and benzoic acid (C_6_H_5_COOH), contain a single carboxylate group and typically act as monodentate ligands, coordinating with individual metal ions.^[^ ^51^ ^]^ These ligands are well suited for the construction of MOFs with smaller pore sizes, contributing to the formation of regular and compact framework structures. This property makes them ideal for applications involving specific molecular screening or catalytic reactions.^[^ ^52^ ^]^ In contrast, polycarboxylic acids, such as terephthalic acid (BDC, C_6_H_4_(COOH)_2_) and trimeric acid (BTC, C_6_H_2_(COOH)_3_), feature two or more carboxylate groups.^[^ ^53^ ^]^ These ligands form multiple coordination bonds with various metal ions, enabling the construction of MOFs with larger pore sizes and more complex network structures.^[^ ^54^ ^]^ For instance, MOFs synthesized with BDC, such as MOF‐5, exhibit exceptional gas adsorption performance, particularly for hydrogen and methane storage, due to the effective coordination of its two carboxylate groups with metal ions.^[^ ^55^ ^]^

The modifiability of carboxylic acid‐based ligands provides a significant advantage, as their functionalization enables tailored adjustments to MOF pore properties and chemical environments.^[^ ^56^ ^]^ For example, introducing functional groups such as amines or halogens can enhance the selectivity of MOFs for specific molecules, improving their effectiveness in applications such as gas adsorption, molecular separation, and catalysis.^[^ ^57^ ^]^ This functionalization not only enhances the chemical diversity of MOFs but also broadens their utility in sensing, drug delivery, and other advanced applications.^[^ ^58^ ^]^ Additionally, carboxylate‐based ligands contribute to the catalytic functionality of MOFs. They can act as active sites or carriers for catalytic reactions, enabling chemical processes such as redox reactions and C—C coupling.^[^ ^59^ ^]^ The ease with which carboxylic acid groups can be modified facilitates postsynthetic functionalization, allowing MOFs to be fine‐tuned for specific catalytic reactions by altering their chemical environment.^[^ ^60^ ^]^ In summary, carboxylate‐based ligands are integral to the design and synthesis of MOFs, offering structural diversity and functionality.^[^ ^61^ ^]^ Through rational design and functionalization, these ligands unlock a broad spectrum of application possibilities, including gas storage, molecular separation, and catalysis. Their versatility underscores their significance in the development of advanced MOF materials for energy storage and conversion technologies.

#### 2.3.2 Imidazole‐Based Ligands

**Figure 3:**
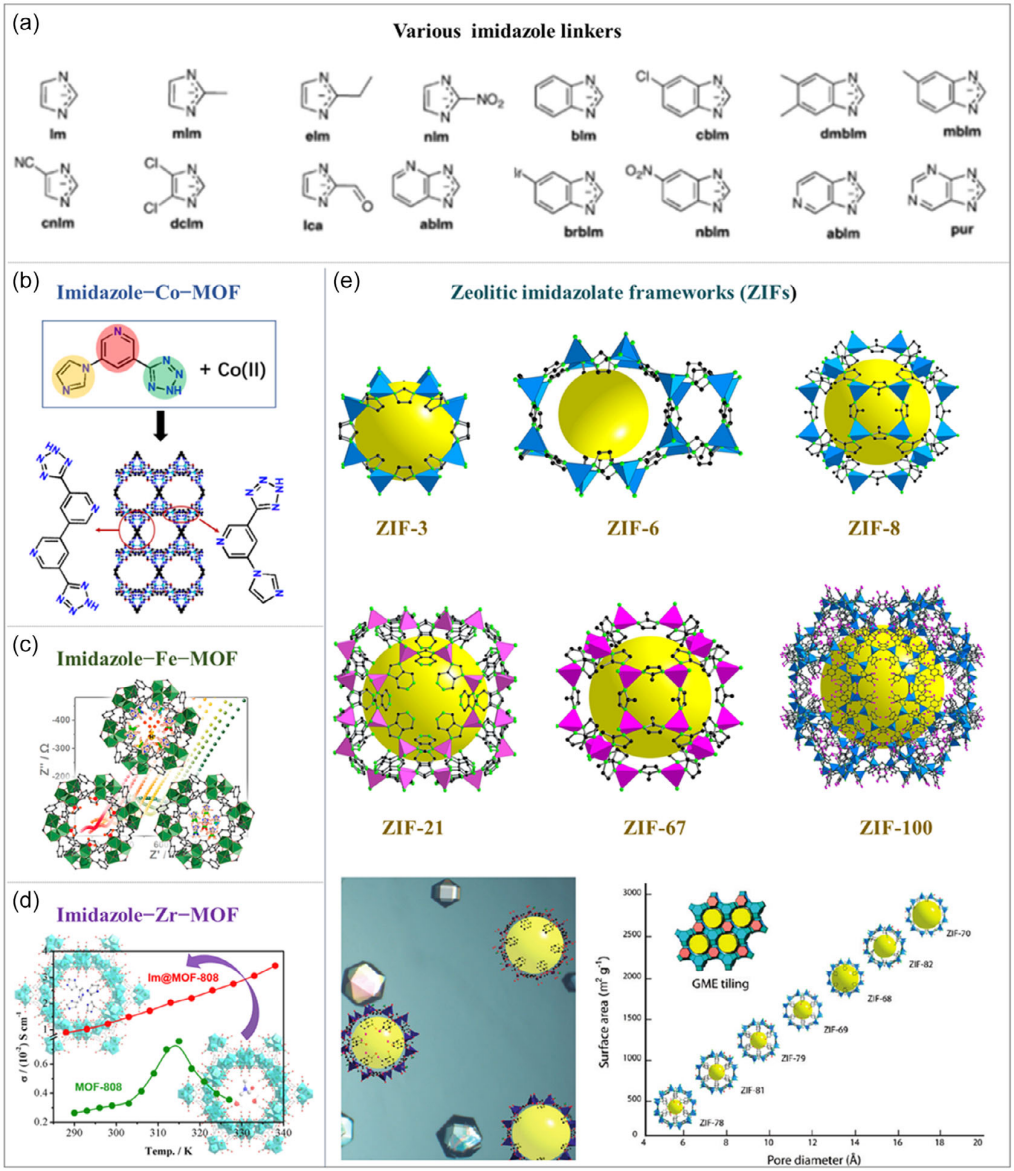
a) Structures of various imidazole linkers for MOFs and the schematic reaction for synthesizing MOFs with imidazole ligands and different metal ions. b) Co^2+^ ions. Reproduced with permission.^[^ ^135^ ^]^ Copyright 2023, American Chemical Society. c) Fe^2+^/Fe^3+^ ions. Reproduced with permission.^[^ ^73^ ^]^ Copyright 2017, American Chemical Society. d) Zr^3+^ ions. Reproduced with permission.^[^ ^136^ ^]^ Copyright 2019, American Chemical Society. e) ZIF: Zeolitic imidazolate framework. Reproduced with permission.^[^ ^137^ ^]^ Copyright 2010, American Chemical Society.

Imidazole‐based ligands play a significant role in the construction and functionality of MOFs due to their core structure, the imidazole ring. This structure imparts unique chemical and physical properties to MOFs, as the nitrogen atoms in the imidazole ring can form stable coordination bonds with metal ions, contributing to the structural framework of MOFs (**Figure** **3**).^[^ ^62^ ^]^ The modifiability of imidazole‐based ligands provides exceptional flexibility for the postfunctionalization of MOFs. By introducing substituents such as halogen, nitro, or amino groups, the electronic properties and coordination characteristics of MOFs can be finely tuned, enhancing their chemical functionality and expanding their application potential in areas such as catalysis.^[^ ^63^ ^]^ The diversity of imidazole‐based ligands arises from their derivatives, such as 1‐methylimidazole or 2‐methylimidazole,^[^ ^64^ ^]^ which carry additional substituents that influence their coordination modes with metal ions and, consequently, the performance of MOFs.^[^ ^65^ ^]^ In particular, zeolitic imidazolate frameworks (ZIFs), composed of 2‐methylimidazole ligands and zinc ions, demonstrate enhanced light absorption and charge separation efficiency when combined with photosensitizers or metal cocatalysts, thereby improving its performance in photocatalytic hydrogen production.^[^ ^66^ ^]^

The versatility of imidazole‐based ligands is further demonstrated by their ability to serve as active sites or carriers of catalysts, enabling MOFs to participate in various chemical reactions, including redox reactions and C—C coupling.^[^ ^67^ ^]^ The nitrogen atoms in the imidazole ring acts as Lewis base sites, facilitating proton transfer and electron transfer during catalytic reaction. Moreover, MOFs with imidazole‐based ligands can enhance hydrogen purity via selectively adsorbing impurities, which is crucial for hydrogen storage and transportation. This ability broadens the scope of imidazole ligands in MOF applications, making them integral to hydrogen energy systems.^[^ ^68^ ^]^

In summary, the structural diversity and modifiability of imidazole‐based ligands provide a broad spectrum of possibilities for tailoring MOFs’ functionality and expanding their application potential. In the context of hydrogen production, MOFs with imidazole ligands play pivotal roles in photoelectrocatalysis for water splitting, as well as in hydrogen storage, purification, and detection.^[^ ^69^ ^]^ These attributes make imidazole ligands indispensable in the design of next‐generation MOF materials for energy and environmental applications.

#### 2.3.3 Triazine‐Based Ligands

**Figure 4:**
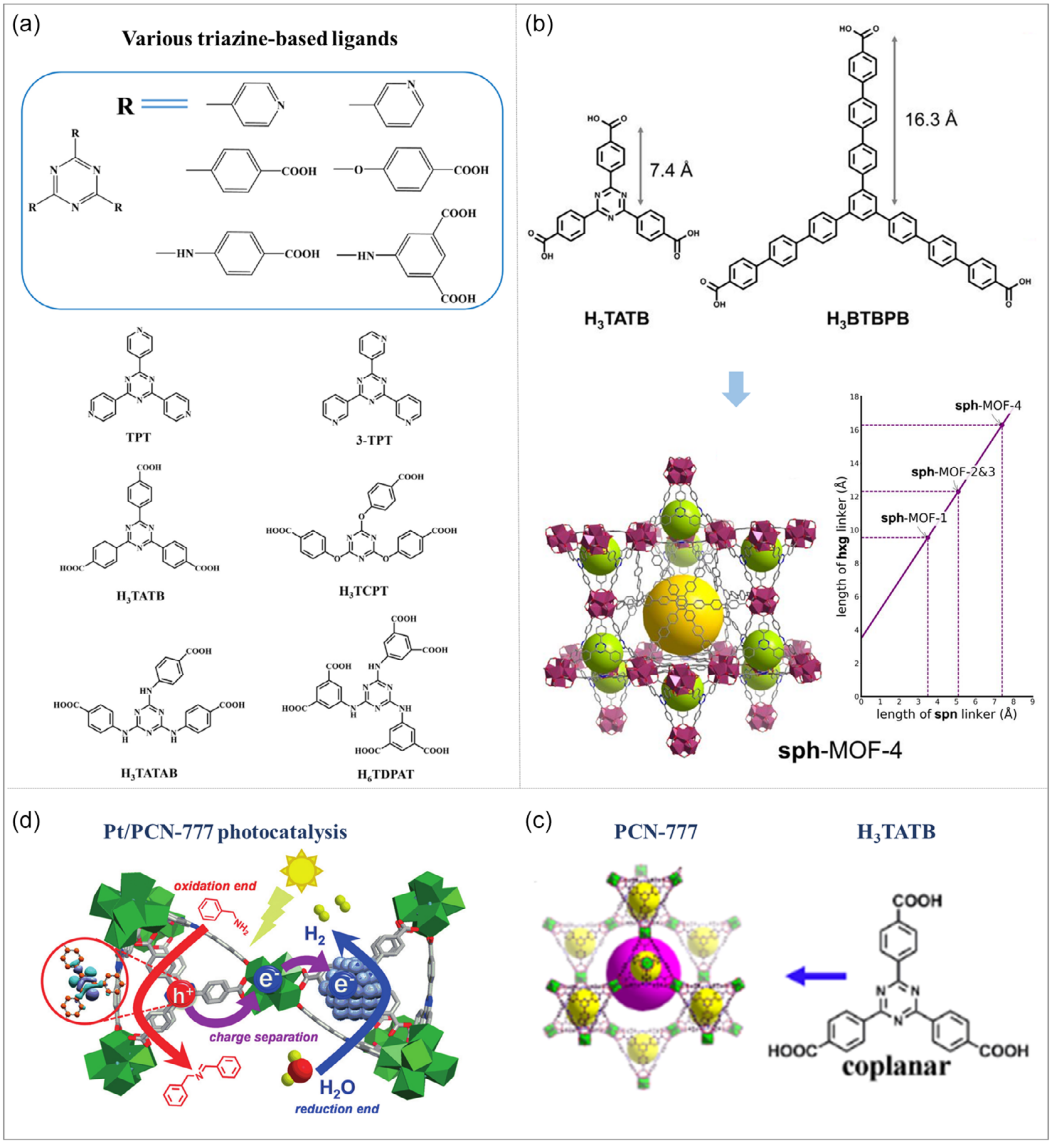
a) Structures of various triazine linkers for MOFs and the schematic reaction for synthesizing MOFs with triazine ligands. b) sph‐MOF‐4. Reproduced with permission.^[^ ^138^ ^]^ Copyright 2018, Royal Society of Chemistry. c) PCN‐777. Reproduced with permission.^[^ ^139^ ^]^ Copyright 2016, American Chemical Society. d) Pt/PCN‐777 photocatalysis. Reproduced with permission.^[^ ^140^ ^]^ Copyright 2018, Wiley‐VCH.

Triazine‐based ligands are characterized by their core triazine ring structure, a six‐membered ring consisting of three carbon atoms and three nitrogen atoms (**Figure** **4**).^[^ ^70^ ^]^ This unique structure provides multiple coordination sites that enable the formation of stable coordination bonds with metal ions, significantly enhancing the structural stability of MOFs.^[^ ^71^ ^]^ In addition, triazine‐based ligands offer the ability to finely tune the electronic band structure and layer flatness of MOFs by modifying the connected N‐heterocyclic species.^[^ ^72^ ^]^ This capability is particularly important for improving the optoelectronic properties of MOFs, such as charge separation and transport efficiency.^[^ ^73^ ^]^ One notable example is PCN‐777, which leverages the properties of the triazine ring to enhance both the structural stability of MOFs and the separation and transfer of photogenerated charges.^[^ ^74^ ^]^ The triazine ring's ability to regulate electronic structures plays a critical role in this process. Upon photoexcitation, electrons on the triazine ring are efficiently transferred to a platinum cocatalyst, where they reduce protons to generate hydrogen. Simultaneously, the holes on the triazine ring oxidize benzamine to form benzamine cation radicals, which are subsequently converted into N‐benzylbenzylideneimine. The triazine group is essential in facilitating charge separation and improving the overall efficiency of the photocatalytic reaction. These attributes enable PCN‐777 to deliver outstanding performance in photocatalytic hydrogen production, showcasing the significant value of triazine‐based ligands in the development of high‐performance MOF materials.

Another example is the UIO‐66‐NH_2_/covalent triazine‐based framework composite, which constructs a type II *Z*‐type heterojunction structure to significantly enhance visible light capture and improve charge transfer separation.^[^ ^38^ ^]^ The incorporation of triazine‐based ligands in this composite structure demonstrates their potential to synergistically enhance photocatalytic activity. In summary, triazine‐based ligands play a pivotal role in the design of MOFs by providing structural stability, electronic tunability, and enhanced optoelectronic properties. Their ability to improve charge separation and facilitate efficient photocatalytic reactions establishes them as key components in the development of advanced MOFs for hydrogen production and related energy applications.

#### 2.3.4 Pyridyl‐Based Ligands

**Figure 5:**
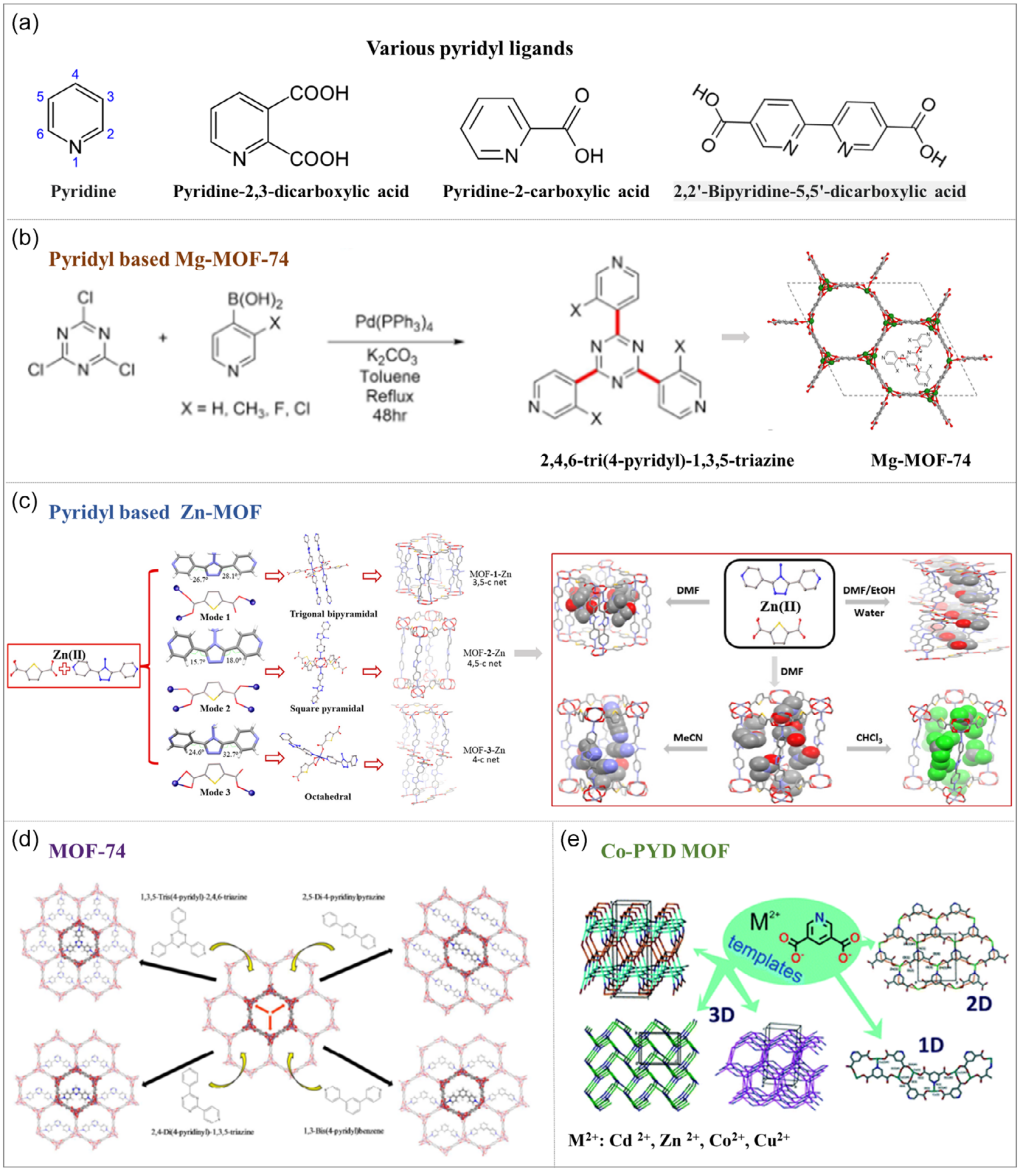
a) Structures of various pyridyl‐based linkers for MOFs and the schematic reaction for synthesizing MOFs with pyridyl‐based ligands. b) Mg‐MOF‐74. Reproduced with permission.^[^ ^141^ ^]^ Copyright 2024, American Chemical Society. c) Zn‐MOF. Reproduced with permission.^[^ ^142^ ^]^ Copyright 2024, American Chemical Society. d) MOF‐74 and e) Co‐PYD MOF. Reproduced with permission.^[^ ^143^ ^]^ Copyright 2018, American Chemical Society; Copyright 2020, Royal Society of Chemistry.

Pyridyl‐based ligands in MOFs are organic molecules featuring a pyridine ring structure, a six‐membered carbon‐nitrogen heterocycle where the nitrogen atoms serve as coordination sites, forming stable coordination bonds with metal ions (**Figure** **5**).^[^ ^75^ ^]^ These ligands provide coordination sites and can impart specific chemical functionalities, such as catalytic activity, to MOFs.^[^ ^76^ ^]^ The diversity and modifiability of pyridyl ligands offer extensive flexibility in designing MOFs with tailored properties. For example, MOF‐74, constructed using pyridyl ligands, features a 3D framework structure with regular pores formed through the coordination of pyridyl rings with metal ions.^[^ ^77^ ^]^ This structure exhibits exceptional gas adsorption and storage capabilities due to its high thermal and chemical stability. MOF‐74 is particularly effective for hydrogen and carbon dioxide adsorption, demonstrating its potential for application in energy storage and environmental management.^[^ ^78^ ^]^

Another example is Co‐PYD (cobalt ion Pyridine‐2,6‐Dicarboxylate), a specially designed pyridyl‐based MOF that exhibits remarkable activity in photocatalytic hydrogen production. In Co‐PYD, the pyridine groups enhance the coordination between MOF and metal nodes, facilitating the separation and transfer of photogenerated charges through their electronic properties, which in turn improves photocatalytic efficiency. This demonstrates the versatility of pyridyl ligands in enhancing MOF functionality for energy conversion and catalysis.

The incorporation of pyridyl‐based multidentate ligands has significantly enriched the chemical functionality and structural diversity of MOFs, expanding their applications in gas storage, molecular separation, chemical sensing, heterogeneous catalysis, and biomedicine. For instance, complex dynamic structures with multiple stimulus–responsive properties can be achieved through the rational design of pyridyl ligands, enabling the development of innovative multiresponsive materials. Moreover, multidentate pyridyl ligands facilitate postsynthetic modifications of MOFs, allowing for performance optimization through advanced strategies such as solvent‐assisted ligand synthesis, atomic layer stacking, or replacement of connecting bridges.

In summary, pyridyl‐based ligands play a crucial role in MOF design, providing structural support and enabling wide‐ranging applications. Their modifiability and electronic properties allow for tailored enhancements in catalysis, energy conversion, and sensing, making them a key component in advancing MOF materials for diverse scientific and industrial applications.

## 3 Synthesis Strategies of TM‐Based MOFs

**Figure 6:**
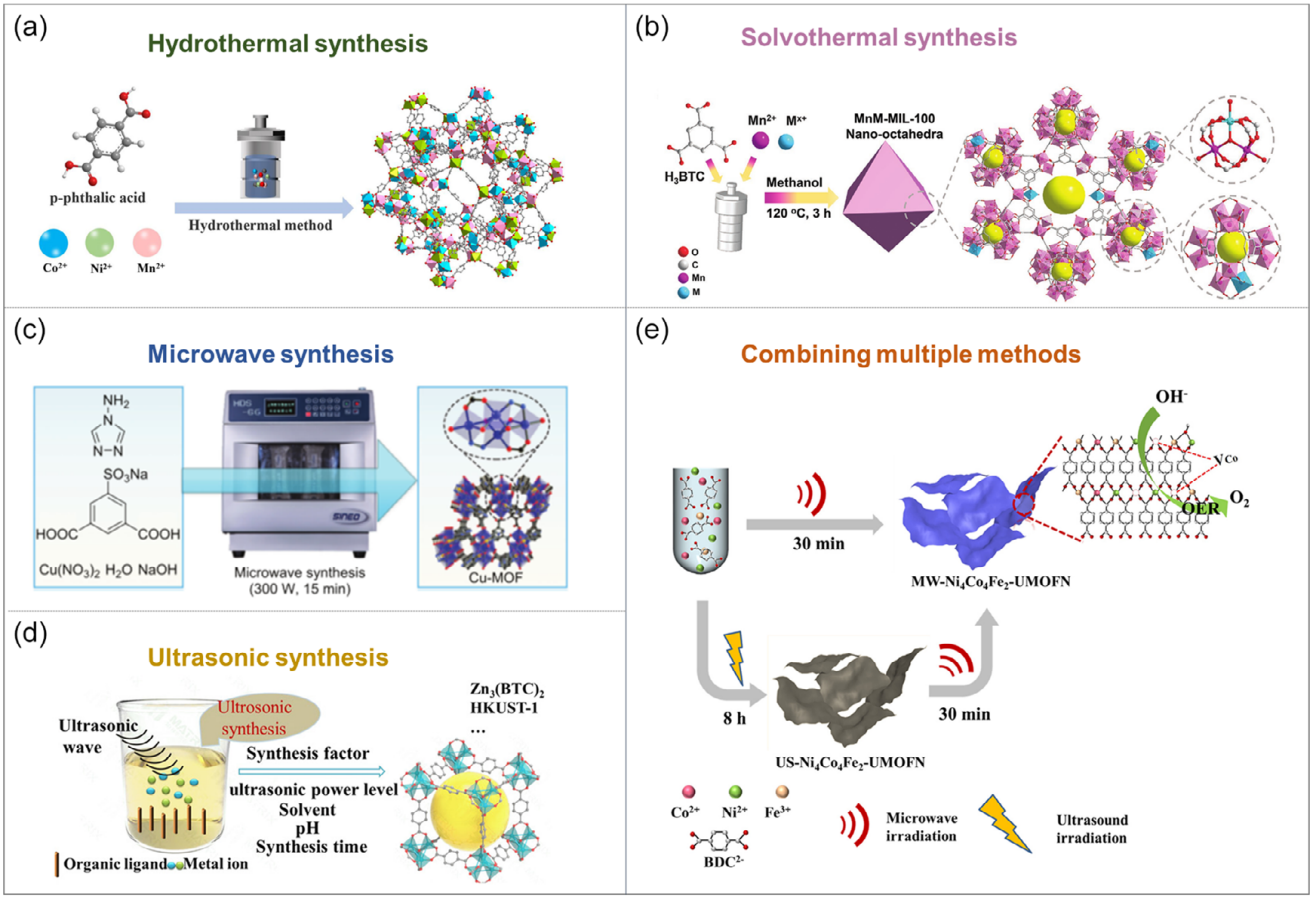
Typical solution‐phase synthetic strategies for MOFs and MOF composites. a) Hydrothermal synthesis. Reproduced with permission.^[^ ^144^ ^]^ Copyright 2021, Springer Nature. b) Solvothermal synthesis. Reproduced with permission.^[^ ^145^ ^]^ Copyright 2021, Wiley‐VCH. c) Microwave‐assisted method. Reproduced with permission.^[^ ^146^ ^]^ Copyright 2021, Wiley‐VCH d) Ultrasound‐assisted method. Reproduced with permission.^[^ ^147^ ^]^ Copyright 2022, Royal Society of Chemistry. e) Combining multiple methods. Reproduced with permission.^[^ ^148^ ^]^ Copyright 2021, Elsevier B.V.

The synthesis strategies for TM‐based MOFs are remarkably diverse (**Figure** **6**), encompassing a wide range of methodologies that integrate traditional and modern technologies to meet various application demands.^[^ ^79^ ^]^ Among the classical approaches, solvothermal and hydrothermal synthesis stand out as foundational methods.^[^ ^80^ ^]^ These techniques leverage high‐temperature and high‐pressure environments to facilitate crystal nucleation and growth, making them particularly suitable for producing TM‐based MOF microcrystals and single crystals with high thermal stability.^[^ ^81^ ^]^ Ultrasonic synthesis accelerates the nucleation and crystallization process through the bubble dynamics effect generated by ultrasound in the solvent, enabling the rapid formation of small‐sized MOF crystals.^[^ ^82^ ^]^ However, challenges such as ensuring structural diversity and maintaining crystal purity require further optimization and precise control. Similarly, microwave‐assisted synthesis uses microwave radiation to quickly heat the reaction system, accelerating chemical reactions and enabling the production of small, high‐phase‐purity TM‐based MOF crystals.^[^ ^83^ ^]^ This method offers rapid heating, high energy conversion efficiency, and is particularly advantageous for rapid synthesis.

Electrochemical synthesis provides a unique method for synthesizing MOFs under mild conditions by leveraging the anode or cathode reactions during electrolysis. This approach facilitates the continuous production of MOFs with controllable particle morphology.^[^ ^84^ ^]^ While the method excels in synthesizing specific MOF types, issues such as low yield and byproduct formation remain significant challenges. Mechanochemical synthesis, which relies on mechanical grinding, reduces solvent usage and overall costs, making it ideal for large‐scale MOF production. Furthermore, this approach allows researchers to directly observe crystal growth, offering deeper insights into the synthesis process.^[^ ^85^ ^]^ Room‐temperature synthesis provides a gentle strategy for fabricating mesoporous MOFs under neutral conditions, enabling the in situ encapsulation of unstable functional molecules.^[^ ^86^ ^]^ This method expands the scope of MOF applications, particularly in scenarios requiring the preservation of sensitive materials. Additional techniques, such as layer‐by‐layer growth, liquid‐phase diffusion, and spray drying, further enrich the synthesis toolbox, each offering distinct advantages depending on the intended application and synthesis conditions.^[^ ^87^ ^]^

The diverse array of MOF synthesis strategies enables researchers to tailor the structural, chemical, and functional properties of MOFs to meet specific application requirements.^[^ ^88^ ^]^ While classical methods like solvothermal and hydrothermal synthesis continue to provide robust and reliable solutions, modern techniques such as microwave‐assisted, ultrasonic, and room‐temperature synthesis offer significant advancements in efficiency and scalability.^[^ ^89^ ^]^ By combining and refining these strategies, researchers can develop MOFs with unprecedented properties, paving the way for their integration into cutting‐edge energy conversion, storage, and environmental technologies.^[^ ^90^ ^]^

### 3.1 Hydrothermal Synthesis

**Figure 7:**
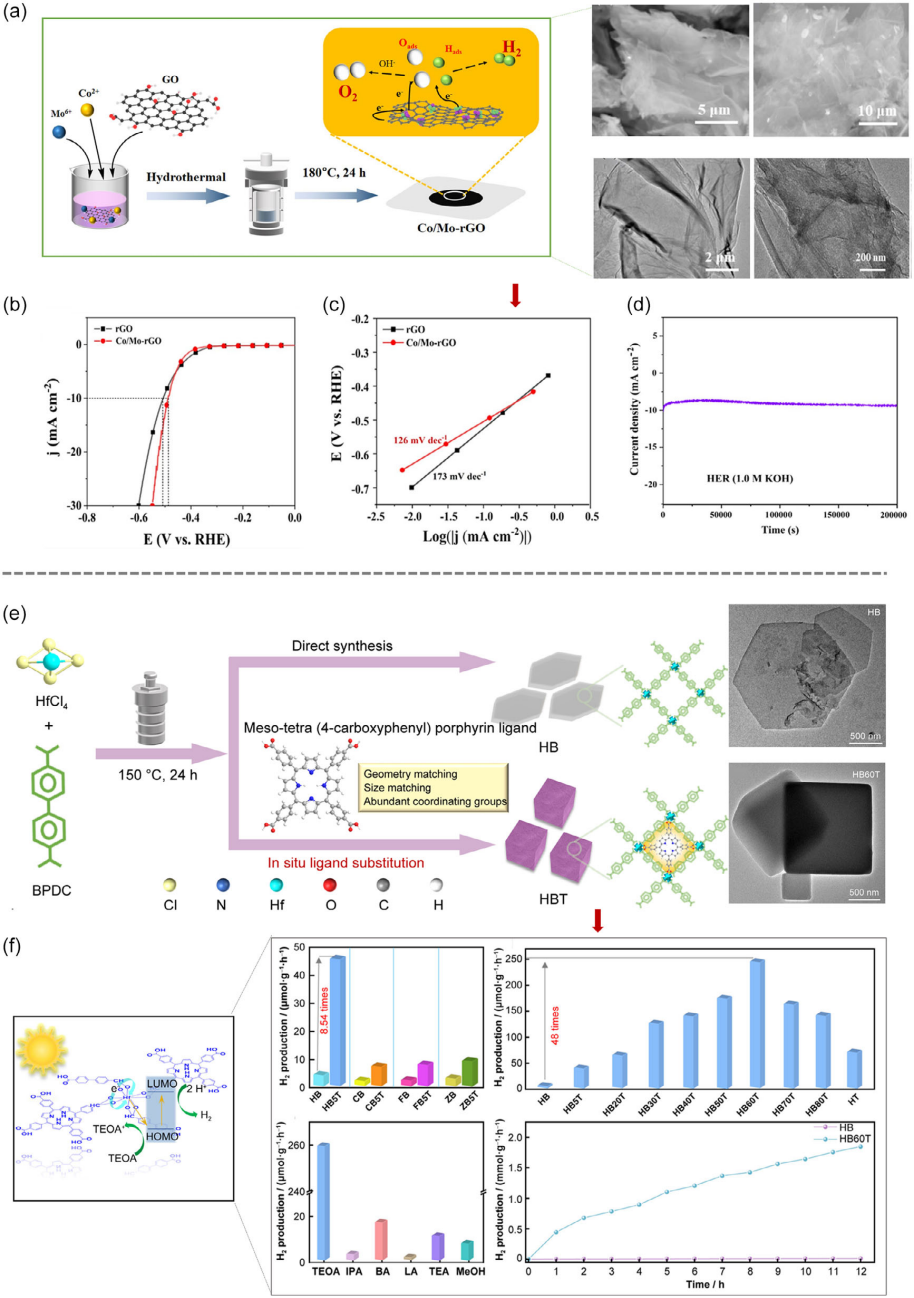
a) Schematic illustration of the hydrothermal strategy for Co/Mo‐MOF. Co/Mo‐MOF HER performance. b) LSV curves, c) Tafel plots, and d) stability tests. Reproduced with permission.^[^ ^149^ ^]^ Copyright 2024, Springer Nature, e) Schematic illustration of the design and f) preparation of HBT (Hf‐BPDC‐TCPP MOF) and HB (Hf‐BPDC MOF) samples with their photocatalytic H_2_ production performance. Hf‐BPDC MOF: Hf‐biphenyl dicarboxylic acid (BPDC) MOF, TCPP: geometry‐matched meso‐tetra (4‐carboxyphenyl) porphyrin ligand. Reproduced with permission.^[^ ^150^ ^]^ Copyright 2024, Springer Nature.

Hydrothermal synthesis is a widely employed method for fabricating MOFs, utilizing water as reaction media under high‐temperature and high‐pressure conditions.^[^ ^91^ ^]^ Typically conducted at temperatures ranging from 100 to 300 °C and pressures between 1 and 100 MPa, this method facilitates the dissolution and reaction of metal salts and organic ligands in a solvent.^[^ ^92^ ^]^ The resulting mixture is heated in a sealed autoclave for a specified duration, promoting controlled crystal nucleation and growth.^[^ ^93^ ^]^ Precise adjustment of reaction parameters, including temperature, pressure, solvent type, reaction time, and reactant concentration, allows for significant customization of the crystal growth process and the final MOF properties (**Figure** **7**).^[^ ^94^ ^]^ The adaptability of hydrothermal synthesis is one of its key strengths, with conditions being tailored to meet specific experimental requirements.^[^ ^95^ ^]^ A standard protocol involves reacting a 0.1 mol zinc nitrate (Zn(NO_3_)_2_) and 0.2 mol 2‐methylimidazole (Hmim) aqueous solution at 150 °C and 15 MPa for 24 h, resulting in regular hexagonal ZIF‐8 crystals. Optimizations, such as increasing the temperature to 180 °C, reducing the Hmim concentration to 0.1 mol, and shortening the reaction time to 12 h, have been shown to produce smaller ZIF‐8 crystals with higher specific surface areas. These optimized crystals exhibit enhanced performance in catalysis and adsorption, underscoring the importance of synthesis condition control.

Hydrothermal synthesis remains a cornerstone technique in MOF production, offering robust control over crystal formation and properties. Its ability to generate materials with tailored characteristics makes it invaluable for advancing applications in catalysis, gas storage, and separation technologies.

### 3.2 Solvothermal Synthesis

**Figure 8:**
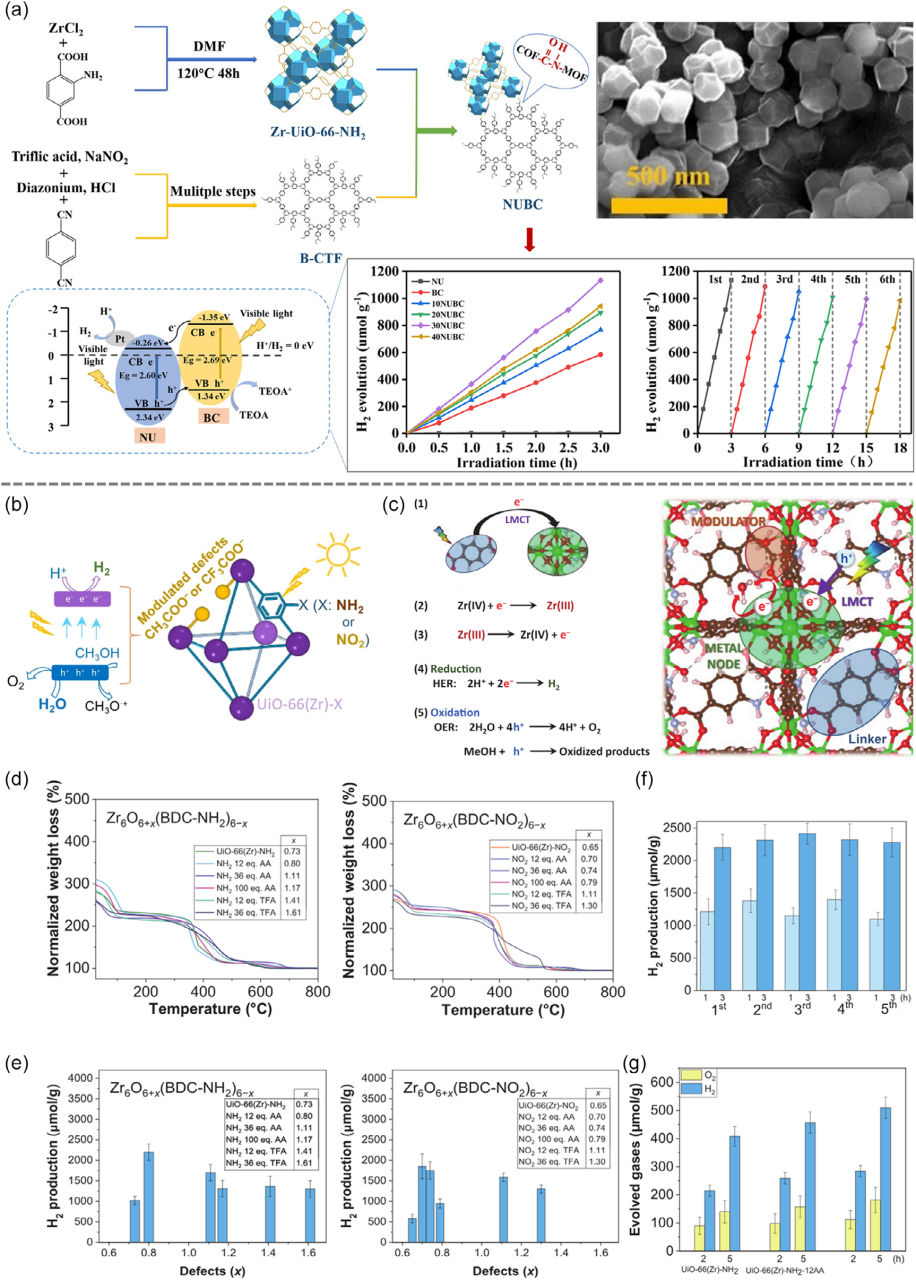
a) Schematic diagram of the synthesis of NUBC hybrid materials, the mechanism of NUBC photocatalytic H_2_ evolution reaction, the stability of NU, BC, and a series of NUBC under light irradiation. NUBC: hybrid materials combining different amounts of Zr‐UiO‐66‐NH_2_ (NU) with a benzoic acid‐modified covalent triazine‐based framework (BC). Reproduced with permission.^[^ ^151^ ^]^ Copyright 2023, Springer Nature. b) Photocatalytic water splitting for H_2_ using UiO‐66(Zr)‐*x* (*X*: NH_2_/NO_2_). c) Plausible photocatalytic reaction mechanism during HER and overall water splitting (OWS) using UiO‐66(Zr)‐NH_2_‐12AA. UiO‐66(Zr)‐NH_2_/UiO‐66(Zr)‐NO_2_ performance. d) TGA analysis. e) Relationship between photocatalytic H_2_ generation and HER. UiO‐66(Zr)‐NH_2_‐12AA photocatalytic H_2_ generation: f) HER and g) OWS. Reproduced with permission.^[^ ^152^ ^]^ Copyright 2024, Springer Nature.

Solvothermal synthesis is a versatile and advanced material synthesis technique that utilizes organic solvents to facilitate the controlled chemical reactions between metal salts and organic ligands, enabling the efficient preparation of MOFs with tailored structures and morphologies (**Figure** **8**).^[^ ^96^ ^]^ Compared to hydrothermal synthesis, solvothermal methods offer greater flexibility in regulating the crystal structure and morphology of MOFs due to the wide range of organic solvents that can be employed.^[^ ^97^ ^]^ These solvents include alcohols, amines, ketones, and other organic compounds capable of dissolving metal salts and ligands, thus providing a broader spectrum of reaction environments.^[^ ^98^ ^]^

The versatility of solvothermal synthesis has been demonstrated in the production of various MOF structures optimized for hydrogen production. For example, Ni‐MOF nanorods synthesized solvothermally exhibit a large specific surface area and an optimized electronic structure, making them highly effective for electrocatalytic hydrogen production.^[^ ^99^ ^]^ The Ni sites in these nanorods act as active centers, effectively promoting the proton reduction reaction. Similarly, Zn‐MOF nanospheres, prepared by hydrothermal synthesis, deliver excellent photocatalytic hydrogen production performance due to their uniform pore structure and high specific surface area, which facilitate the separation and transfer of photogenerated charge carriers.^[^ ^100^ ^]^ Solvothermal synthesis has also enabled the creation of Cu‐MOF nanosheets, characterized by abundant active sites and enhanced charge transport performance. These properties contribute to their exceptional activity and stability in electrocatalytic hydrogen production.^[^ ^101^ ^]^ Meanwhile, Co‐MOF hollow spheres prepared by hydrothermal synthesis leverage their unique hollow structure and high porosity to boost photocatalytic hydrogen production efficiency by increasing light absorption through internally reflected photons.^[^ ^102^ ^]^ Further innovations include Fe‐MOF dendritic structures synthesized solvothermally. These dendrites feature increased surface area and porosity, providing additional active sites and optimized electron transport pathways, which are critical for improving electrocatalytic hydrogen production.^[^ ^103^ ^]^ Similarly, Zr‐MOFs, produced via hydrothermal methods, exhibit adjustable interlayer spacing that facilitates the separation and transmission of photogenerated charge carriers, significantly enhancing their photocatalytic performance.^[^ ^104^ ^]^

In summary, solvothermal synthesis offers a powerful approach for fabricating MOFs with diverse and application‐specific morphologies. Its ability to adapt reaction environments through the use of various solvents allows for precise control over the structure and properties of MOFs, making it indispensable in the development of advanced materials for hydrogen production and other energy‐related applications.

### 3.3 Microwave Synthesis

Microwave synthesis is a cutting‐edge technique that uses microwave radiation as an energy source to promote rapid coordination reactions between metal salts and organic ligands, facilitating the efficient synthesis of MOFs.^[^ ^105^ ^]^ The unique volumetric heating mechanism of microwaves ensures that all molecules in the reaction system are heated uniformly and simultaneously.^[^ ^106^ ^]^ This approach bypasses the limitations of traditional heating methods, such as heat conduction and convection, leading to significantly accelerated reaction rates and shorter synthesis cycles. For instance, microwave‐assisted synthesis has been employed to optimize the production of zirconium‐based MOFs such as UiO‐66.^[^ ^107^ ^]^ By adjusting the amount of modulator used, researchers have successfully enhanced the yield and porous properties of the product, achieving a high specific surface area (907.63 m^2^ g^−1^) and substantial pore volume within just a few seconds of reaction time.^[^ ^108^ ^]^ This contrasts sharply with traditional solvothermal methods, which often require hours to achieve comparable results.

Innovative strategies leveraging microwave synthesis have further expanded its application scope. For example, using tin dioxide (SnO_2_) as a linker, a two‐step microwave‐assisted chemical modification process has been developed to immobilize single‐atom catalysts, such as platinum (Pt), copper (Cu), and nickel (Ni), onto MOF carriers like UiO‐66‐NH_2_, DUT‐67, and PCN‐222.^[^ ^109^ ^]^ This method not only accelerates the chemical reaction rate but also enhances the interaction between the single‐atom catalysts and MOF carriers, thereby improving the structural stability and catalytic performance of the resulting materials. Notably, the Pt_1_/SnO_2_/UiO‐66‐NH_2_ catalyst exhibits excellent photocatalytic hydrogen production efficiency under visible light, significantly outperforming traditional catalysts and MOFs without single‐atom loading. In addition to its superior catalytic efficiency, the Pt_1_/SnO_2_/UiO‐66‐NH_2_ catalyst exhibits cycle stability maintaining its performance after three consecutive catalytic cycles. This long‐term stability and reliability are crucial for practical applications. Structural characterization and theoretical calculations confirm the uniform dispersion of Pt single atoms within the catalyst and highlight the synergistic effects between the single‐atom catalysts and MOF carriers. These findings provide robust theoretical and experimental support for developing next‐generation photocatalytic hydrogen production catalysts.^[^ ^108^ ^]^

Microwave synthesis thus offers a highly efficient, rapid, and versatile approach for MOF production. Its ability to fine tune material properties and incorporate advanced functionalities, such as single‐atom catalysts, underscores its potential to drive innovation in MOF‐based applications, particularly in energy conversion and hydrogen production.

### 3.4 Ultrasonic Synthesis

Ultrasonic synthesis is an innovative and efficient method for preparing MOFs, leveraging ultrasonic waves to drive the nucleation and crystallization processes.^[^ ^110^ ^]^ In this technique, raw materials are dissolved in a solvent, and ultrasonic waves are applied to create cavitation bubbles. The cycles of bubble formation, growth, and collapse generate localized high temperatures and pressures, promoting uniform nucleation, reducing crystallization time, and aiding in the formation of smaller crystals.^[^ ^111^ ^]^ Compared to traditional solvothermal methods, ultrasonic‐assisted synthesis significantly shortens synthesis time and yields MOF particles with smaller sizes, potentially increasing surface area and porosity, which enhance the functionality of the resulting materials.^[^ ^112^ ^]^

The synthesis process can be fine tuned by adjusting the time and power of ultrasonic treatment, providing precise control over the morphology and size of MOF particles.^[^ ^113^ ^]^ For example, a study on the ultrasound‐assisted synthesis of UiO‐66 demonstrated that this method produces smaller particles (56–155 nm) compared to the traditional solvothermal approach, which yielded an average size of 192 nm.^[^ ^114^ ^]^ The reduction in particle size highlights the ability of ultrasonic synthesis to shorten induction times and influence particle growth by promoting nucleation. These advantages are particularly beneficial for applications requiring high surface area and tunable porosity.

Ultrasonic synthesis also offers the flexibility of being conducted under milder conditions, further contributing to its appeal. MOFs synthesized using this method have shown significant promise in applications such as gas–liquid separation and purification membranes, where enhanced surface area and controlled particle morphology are critical. While ultrasonic synthesis may result in variations in structural purity due to its rapid and dynamic crystallization process, these challenges can be mitigated by optimizing ultrasonic parameters such as treatment time and power. In summary, ultrasonic synthesis stands out for its rapidity, controllability, and adaptability, making it a valuable tool for the efficient production of MOFs with tailored properties. Its ability to produce materials with enhanced surface area and porosity positions it as an essential technique in advancing MOF applications in separation, purification, and other functional material domains.

## 4 Application of TM‐Based MOFs in Hydrogen Production

TM‐based MOFs possess remarkable application potential in catalytic hydrogen production due to their high specific surface area, adjustable pore structure, abundant active sites, and tunable chemical compositions. These characteristics make TM‐based MOFs highly advantageous for both electrocatalytic and photocatalytic hydrogen production, enabling efficient and sustainable energy conversion processes.

### 4.1 Electrocatalytic Hydrogen Production

TM‐based MOFs are highly emerging as promising electrocatalysts for hydrogen production reactions due to their unique physicochemical properties, including high specific surface area, tunable porosity, and diverse active sites.^[^ ^115^ ^]^ Electrocatalytic hydrogen production involves two fundamental electrochemical processes: the HER, where protons are reduced to hydrogen, and the OER, which oxidizes water molecules to oxygen and protons.^[^ ^116^ ^]^ The efficiency and selectivity of these two reactions largely depend on the performance of the electrocatalyst. TM‐based MOFs address this challenge by providing abundant active sites and facilitating reactant transport through their high specific surface area and adjustable pore environments.

In the HER, TM‐based MOFs enhance hydrogen generation by offering catalytic active sites. Modifications to the pore environment and structural elements with intrinsic catalytic activity have been shown to significantly improve their HER performance.^[^ ^117^ ^]^ For instance, TM‐based MOFs with bimetallic active sites exploit the synergistic effects between different metal centers to boost electrocatalytic activity.^[^ ^93^ ^]^ TM‐based MOFs also demonstrate potential in OER electrocatalysis. For example, a NiRu_0.08_‐MOF doped with atomically monodispersed Ru exhibited outstanding OER performance, requiring an overpotential of only 187 mV at a current density of 10 mA cm^−^^2^, and showed excellent stability for over 300 h.^[^ ^118^ ^]^ The incorporation of noble metals like Ru optimizes the electronic structure of MOFs, enhancing their catalytic efficiency. Additionally, MOF‐derived metal oxide heterojunctions have shown excellent performance, further illustrating the versatility of MOFs in electrocatalysis and their potential for photocatalytic hydrogen production.^[^ ^119^ ^]^

**Figure 9:**
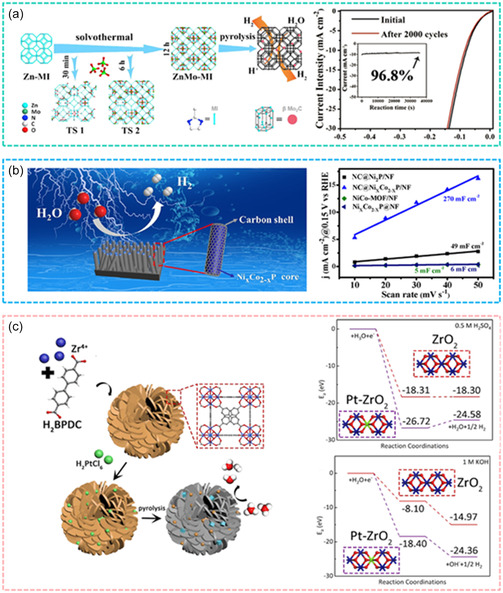
TM‐based MOFs or its nanocomposites for electrocatalytic hydrogen production. a) ZnMo‐MOF‐derived Mo_2_C in N‐doped Catalyst. Reproduced with permission.^[121a]^ Copyright 2021, Elsevier B.V., b) TM‐based MOF nanocomposite for HER electrocatalyst. Reproduced with permission.^[121b]^ Copyright 2021, Elsevier B.V. c) TM‐based MOF carbon electrocatalyst for enhanced HER. Reproduced with permission.^[^ ^122^ ^]^ Copyright 2022, American Chemical Society.

Despite their promise, the labile metal‐organic coordination bonds in TM‐based MOFs can undergo structural reconstruction under electrochemical conditions, potentially compromising their electrocatalytic stability and activity. The electrochemical stability of MOFs, influenced by their chemical composition and electrolyte environment, is a critical determinant of their performance.^[^ ^120^ ^]^ In some cases, MOFs act as precursors, transforming into more active phases, such as metal oxides or hydroxides, during electrochemical reactions. These derived phases actively engage in hydrogen production and often outperform their MOF precursors.^[^ ^120^ ^]^ To overcome these limitations, researchers have developed MOF‐derived nanomaterials and nanocomposites. These advanced materials retain the porous structure and metal node characteristics of their precursors while incorporating new phases, such as metal oxides or carbon materials, to enhance conductivity and catalytic activity. The unique structural and compositional features of these MOF‐derived materials result in superior electrocatalytic performance, providing increased active sites and facilitating mass transfer, which significantly boosts reaction efficiency (**Figure** **9**a,b).^[^ ^121^ ^]^

The high porosity and specific surface area of MOF‐derived nanomaterials create an optimal microenvironment for electrocatalytic reactions, accelerating reaction rates. Moreover, strategic design of MOF precursors allows for the optimization of their derived materials’ electrocatalytic performance, achieving low overpotentials and high electrochemical stability (Figure 9c).^[^ ^122^ ^]^ TM‐based MOFs and their derivatives, with features such as heteroatom doping and optimized electronic structures, exemplify this potential by delivering excellent HER and OER performance.

**Figure 10:**
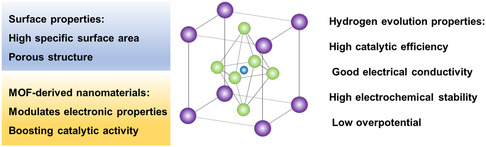
TM‐based MOFs or its nanocomposites with the hydrogen evolution properties.

In practical applications, the catalytic performance of MOF materials has been extensively validated through electrochemical testing and in situ characterization techniques. These evaluations highlight the remarkable potential of MOFs for hydrogen production under pyrolysis conditions, further reinforcing their utility in real‐world scenarios.^[^ ^123^ ^]^ TM‐based MOFs, characterized by their high specific surface area, porous structures, and heteroatom doping, exhibit significant advantages in electrocatalytic hydrogen evolution. These features provide a high density of active sites and enable optimized electronic structures, which facilitate enhanced catalytic activity while maintaining low overpotentials and high electrochemical stability. Importantly, TM‐based MOFs present a cost‐effective alternative to noble metal catalysts, making them a promising candidate for scalable and sustainable hydrogen production technologies (**Figure** **10**). The application of TM‐based MOFs and MOF‐derived materials in electrocatalytic hydrogen production has demonstrated significant advantages, including high catalytic activity, stability, and tunable active sites. With continuous advancements in MOF synthesis and derivation technologies, these materials are poised to play a critical role in the development of efficient and sustainable electrocatalysts for hydrogen production. Their versatility and potential for optimization make MOFs an essential component in the future of energy conversion and storage technologies.

### 4.2 Photocatalytic Hydrogen Production

**Figure 11:**
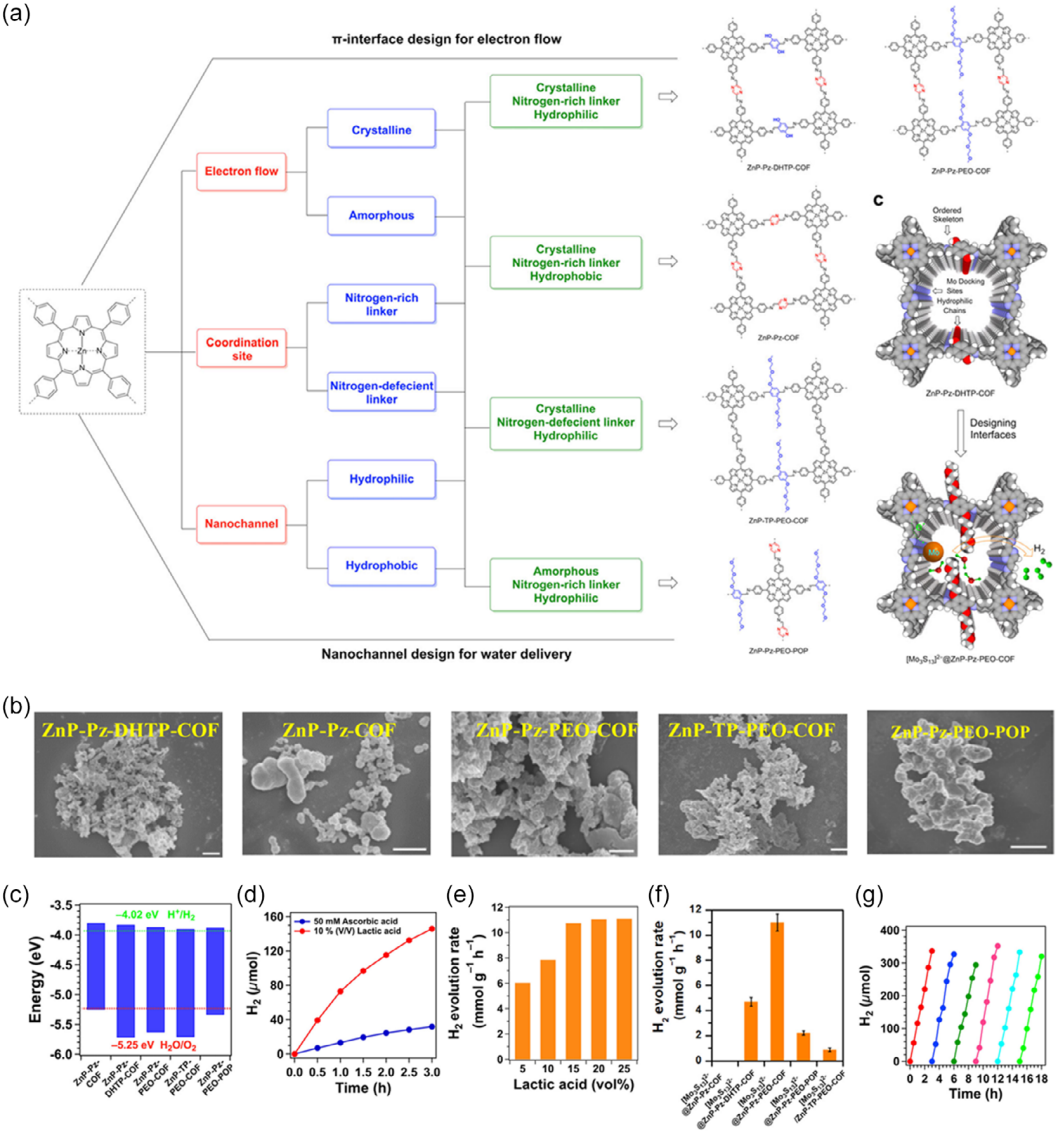
Zn‐MOF for photocatalytic hydrogen production.^[^ ^125^ ^]^ Reproduced with permission. Copyright 2023, Nature Com. a) Integrated interfacial designs of photocatalysts. b) Scanning electron microscope images of Zn‐MOF. Optoelectronic property and photocatalytic activity of Zn‐MOF. c) Time‐dependent hydrogen production profiles monitored. Hydrogen evolution d) with different lactic acid concentrations, e) compared with other analogues (error bars are calculated based on three independent experiments), f) different kinds of covalent organic frameworks (COFs), and g) stability of [Mo_3_S,]^2^@ZnP‐Pz‐PEO‐COF.

The application of TM‐based MOFs in photocatalytic hydrogen production leverages their distinctive structural and functional properties to achieve efficient and sustainable energy conversion.^[^ ^124^ ^]^ The high specific surface area and tunable pore structure of TM‐based MOFs provide abundant active sites for photocatalytic reactions. These structural features also facilitate material transport and ensure effective interaction between reactants and catalysts, making MOFs highly advantageous for photocatalytic applications (**Figure** **11**).^[^ ^125^ ^]^

During the photocatalytic process, the metal nodes or organic ligands in TM‐based MOFs absorb photons from sunlight, triggering the transition of electrons from the valence band to the conduction band. This generates electron–hole pairs, and the effective separation and migration of these carriers to the MOF surface are critical for efficient photocatalysis.^[^ ^126^ ^]^ Electrons reduce protons to produce hydrogen, while holes oxidize water molecules, releasing oxygen. This well‐coordinated mechanism demonstrates the strong potential of MOFs in photocatalytic hydrogen production.

In practical applications, TM‐based MOFs have shown impressive performance improvements through various structural and compositional optimizations. For instance, Pt‐loaded Ti‐MOF‐NH_2_ exhibits significantly enhanced photocatalytic hydrogen production activity due to the synergistic interaction between metal nodes and organic ligands, which improves charge separation and provides more active sites.^[^ ^127^ ^]^ Similarly, heterostructures composed of MOF‐derived metal oxides and 2D ZnIn_2_S_4_ layers exhibit superior catalytic hydrogen production rates compared to their individual components. These enhancements are attributed to improved light absorption, efficient charge separation, and the creation of additional active sites.^[^ ^128^ ^]^ The optimization of TM‐based MOF photocatalytic performance can be achieved through several strategies, including metal doping, ligand functionalization, heterostructure construction, and defect engineering. Metal doping adjusts electronic structures to improve carrier mobility, while functionalizing ligands enhances light absorption and catalytic activity. Constructing heterostructures combines the advantages of MOFs with other materials to improve charge separation and expand light absorption ranges. Defect engineering introduces structural irregularities that create additional active sites and enhance charge transport.

**Figure 12:**
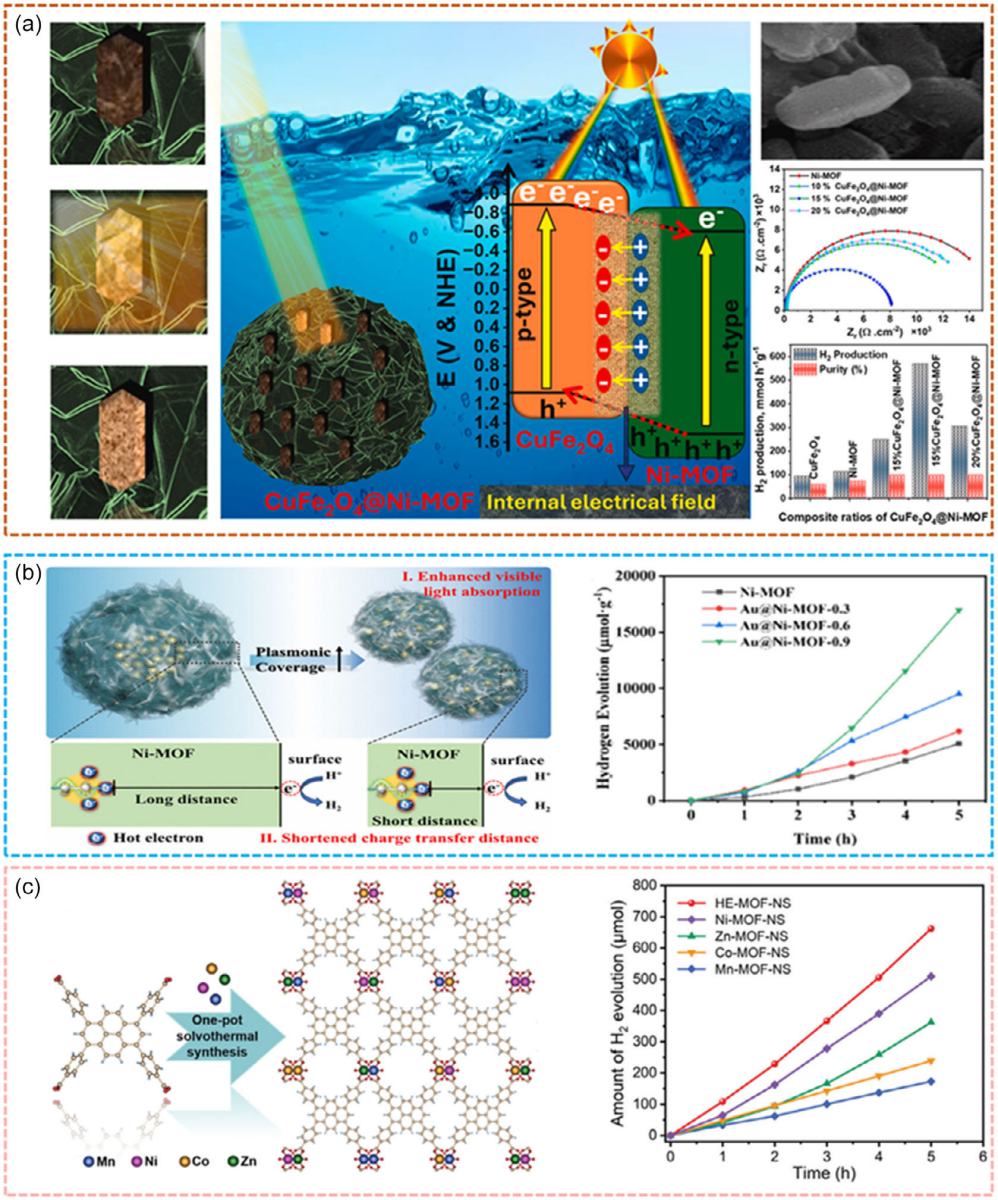
TM‐based MOFs or its nanocomposites for photocatalytic hydrogen production. a) CuFe_2_O_4_@Ni‐MOF photocatalyst for enhanced H_2_ production. Reproduced with permission.^[^ ^129^ ^]^ Copyright 2025, Elsevier B.V. b) Au@Ni‐MOF Core‐Shell photocatalysts for enhanced H_2_ evolution. Reproduced with permission.^[^ ^130^ ^]^ Copyright 2024, American Chemical Society c) H_4_TBAPy unit and 2D double layer for photocatalytic H_2_ evolution. Reproduced with permission.^[^ ^131^ ^]^ Copyright 2024, Wiley‐VCH.

Innovative designs further highlight the versatility of MOFs in photocatalysis. Magnetic MOFs like CuFe_2_O_4_@Ni‐MOF utilize their magnetic properties to enhance catalyst dispersion and light absorption, improving photocatalytic activity (**Figure** **12**a).^[^ ^129^ ^]^ Core–shell‐structured MOFs, such as Au@Ni‐MOF, exploit plasmonic effects to extend visible light absorption and facilitate charge separation, crucial for efficient hydrogen generation (Figure 12b).^[^ ^130^ ^]^ Additionally, high‐entropy MOFs (Figure 12c),^[^ ^131^ ^]^ incorporating multiple metal nodes, enhance charge transfer efficiency through complex electronic environments, further boosting overall performance. These features collectively demonstrate how the rational design of MOFs can maximize light absorption, improve charge transfer, and increase the availability of active sites, all critical for advancing photocatalytic hydrogen production technologies.

In summary, the structural flexibility of MOFs allows for continuous advancements in their photocatalytic capabilities, positioning them as key materials for sustainable energy solutions. Through targeted design and optimization, MOFs can play a pivotal role in achieving efficient and scalable hydrogen production, demonstrating their immense potential in addressing global energy challenges.

## 5 Conclusions and Future Perspectives

TM‐based MOFs have garnered significant attention for their substantial potential in hydrogen production. Their high specific surface area, tunable pore structures, and abundant active sites provide favorable conditions for both electrocatalytic and photocatalytic hydrogen production. These structural advantages enable enhanced material transport and interaction, positioning TM‐based MOFs as promising candidates for energy conversion and catalytic applications. Furthermore, the structural diversity and chemical tunability of TM‐based MOFs allow for the design and synthesis of highly stable and conductive materials, broadening their potential in various catalytic and energy technologies.

Despite their advantages, TM‐based MOFs face challenges in practical applications, particularly concerning their stability and conductivity. The inherently low conductivity of many TM‐based MOFs hinders efficient electron transfer, while their limited chemical and thermal stability restricts their use under harsh reaction conditions. To address these limitations, researchers are pursuing strategies such as designing 2D conductive TM‐based MOFs, introducing unsaturated metal sites, and integrating MOFs with other active components to enhance both conductivity and stability. Precise control over TM‐based MOF microstructures, including their morphology, size, and distribution, is crucial for optimizing charge transport and catalytic performance. Combining TM‐based MOFs with other nanomaterials can further enhance synergistic effects, improving overall efficiency.

Future research directions will focus on developing economical, environmentally friendly, and durable MOF‐based electrocatalysts. Additionally, exploring new catalytic reactions and applications for TM‐based MOFs will remain a priority. Challenges such as cost‐effectiveness, large‐scale production, and long‐term stability must be addressed to achieve broader industrial applications. Investigating regeneration methods and recycling strategies for MOF‐based catalysts will also be critical for ensuring sustainability and reducing material waste.

In conclusion, TM‐based MOFs hold immense promise as multifunctional materials for hydrogen production. Their versatility, combined with continuous advancements in material innovation and performance optimization, is expected to pave the way for more efficient and scalable hydrogen energy technologies. By overcoming existing challenges and leveraging their unique properties, TM‐based MOFs are poised to play an increasingly pivotal role in the future of energy conversion and catalytic technologies, contributing to the global transition toward sustainable energy solutions.

## Conflict of Interest

The authors declare no conflict of interest.

## Author Contributions

**Ting Yang**: conceptualization (lead); validation (lead); visualization (lead); writing—original draft (lead); writing—review & editing (lead). **Hua Zhang**: writing—original draft (supporting); writing—review & editing (equal). **Bo Pang**: supervision (supporting); visualization (supporting); writing—review & editing (supporting). **Jonathan W. C. Wong**: funding acquisition (supporting); writing—original draft (supporting); writing—review & editing (supporting). **Ting Yang** and **Hua Zhang** contributed equally to this work.

## References

[1] S. Evro , B. A. Oni , O. S. Tomomewo , Int. J. Hydrogen Energy 2024, 78, 1449.

[2] S. Kumar , T. Baalisampang , E. Arzaghi , V. Garaniya , R. Abbassi , F. Salehi , J. Cleaner Prod. 2023, 384, 135545.

[3] L. Zhang , C. Jia , F. Bai , W. Wang , S. An , K. Zhao , Z. Li , J. Li , H. Sun , Fuel 2024, 355, 129455.

[4] A. M. Sadeq , R. Z. Homod , A. K. Hussein , H. Togun , A. Mahmoodi , H. F. Isleem , A. R. Patil , A. H. Moghaddam , Sci. Total Environ. 2024, 939, 173622. 38821273 10.1016/j.scitotenv.2024.173622

[5] S. A. H. S. Mousavi , A. H. S. Dehaghani , Energy Convers. Manage. 2024, 299, 117825.

[6] S. Ga , N. An , G. Y. Lee , C. Joo , J. Kim , Renewable Sustainable Energy Rev. 2024, 192, 114275.

[7] B. Zhu , R. Zou , Q. Xu , Adv. Energy Mater. 2018, 8, 1801193.

[8] U. Khan , A. Nairan , J. Gao , Q. Zhang , Small Struct. 2022, 4, 2200109.

[9] J. Gao , Q. Huang , Y. Wu , Y.‐Q. Lan , B. Chen , Adv. Energy Sustainability Res. 2021, 2, 2100033.

[10] V.‐A. Thai , T.‐B. Nguyen , C.‐W. Chen , X.‐T. Bui , R. Doong , C.‐D. Dong , Environ. Sci.: Nano 2024, 11, 3871.

[11] A. Hanan , M. N. Lakhan , F. Bibi , A. Khan , I. A. Soomro , A. Hussain , U. Aftab , Chem. Eng. J. 2024, 482, 148776.

[12] S. Zheng , X. Li , B. Yan , Q. Hu , Y. Xu , X. Xiao , H. Xue , H. Pang , Adv. Energy Mater. 2017, 7, 1602733.

[13] a) T. Devic , C. Serre , Chem. Soc. Rev. 2014, 43, 6097; 24947910 10.1039/c4cs00081a

[14] S. Li , Y. Gao , N. Li , L. Ge , X. Bu , P. Feng , Energy Environ. Sci. 2021, 14, 1897.

[15] Q. Liang , J. Chen , F. Wang , Y. Li , Coord. Chem. Rev. 2020, 424, 213488.

[16] T. Xiao , D. Liu , Microporous Mesoporous Mater. 2019, 283, 88.

[17] J. Morales‐Vidal , R. García‐Muelas , M. A. Ortuño , Catal. Sci. Technol. 2021, 11, 1443.

[18] H. Jiang , Z. Zhao , G. Li , M. Wang , P. Chen , X. Liu , X. Tu , Y. Hu , Z. Shen , Y. Wu , Adv. Sci. (Weinheim, Ger.) 2024, 11, 2306919. 10.1002/advs.202306919 PMC10787075 37985793

[19] R. Wei , C. A. Gaggioli , G. Li , T. Islamoglu , Z. Zhang , P. Yu , O. K. Farha , C. J. Cramer , L. Gagliardi , D. Yang , B. C. Gates , Chem. Mater. 2019, 31, 1655.

[20] F. A. Paz , J. Klinowski , S. M. Vilela , J. P. Tome , J. A. Cavaleiro , J. Rocha , Chem. Soc. Rev. 2012, 41, 1088. 21918788 10.1039/c1cs15055c

[21] Q. Li , W. Yang , F. Li , A. Cui , J. Hong , Int. J. Hydrogen Energy 2018, 43, 271.

[22] Y. Yang , Y. Yang , Y. Liu , S. Zhao , Z. Tang , Small Sci. 2021, 1, 2170030. 10.1002/smsc.202100015 PMC11936017 40212851

[23] H. Zhong , M. Wang , G. Chen , R. Dong , X. Feng , ACS Nano 2022, 16, 1759. 35049290 10.1021/acsnano.1c10544

[24] P. Wang , X. Li , P. Zhang , X. Zhang , Y. Shen , B. Zheng , J. Wu , S. Li , Y. Fu , W. Zhang , F. Huo , ACS Appl. Mater. Interfaces 2020, 12, 23968. 32343548 10.1021/acsami.0c04606

[25] R. Madhu , A. Karmakar , K. Bera , S. Nagappan , H. N. Dhandapani , A. De , S. S. Roy , S. Kundu , Mater. Chem. Front. 2023, 7, 2120.

[26] Y. Shui , N. Deng , Y. Wang , G. Wang , H. Chi , Q. Zeng , Z. Peng , B. Cheng , W. Kang , J. Mater. Chem. A 2024, 12, 20655.

[27] S. Diyali , N. Diyali , B. Biswas , Coord. Chem. Rev. 2024, 500, 215496.

[28] H. C. Zhou , S. Kitagawa , Chem. Soc. Rev. 2014, 43, 5415. 25011480 10.1039/c4cs90059f

[29] H. Y. Li , X. J. Kong , S. D. Han , J. Pang , T. He , G. M. Wang , X. H. Bu , Chem. Soc. Rev. 2024, 53, 5626. 38655667 10.1039/d3cs00873h

[30] M. Y. Masoomi , A. Morsali , A. Dhakshinamoorthy , H. Garcia , Angew. Chem., Int. Ed. Engl. 2019, 58, 15188. 30977953 10.1002/anie.201902229

[31] A. Radwan , H. Jin , D. He , S. Mu , Nanomicro Lett. 2021, 13, 132. 34138365 10.1007/s40820-021-00656-w PMC8169752

[32] a) D. Wang , H. Yao , J. Ye , Y. Gao , H. Cong , B. Yu , Small 2024, 20, 2404350; 10.1002/smll.202404350 39149999

[33] C.‐Y. Hsu , W.‐T. Chung , T.‐M. Lin , R.‐X. Yang , S. S. Chen , K. C. W. Wu , Int. J. Hydrogen Energy 2024, 49, 873.

[34] X. Zhang , Z. Chen , X. Liu , S. L. Hanna , X. Wang , R. Taheri‐Ledari , A. Maleki , P. Li , O. K. Farha , Chem. Soc. Rev. 2020, 49, 7406. 32955065 10.1039/d0cs00997k

[35] S. Patial , P. Raizada , V. Hasija , P. Singh , V. K. Thakur , V. H. Nguyen , Mater. Today Energy 2021, 19, 100589.

[36] a) R. Freund , O. Zaremba , G. Arnauts , R. Ameloot , G. Skorupskii , M. Dinca , A. Bavykina , J. Gascon , A. Ejsmont , J. Goscianska , M. Kalmutzki , U. Lachelt , E. Ploetz , C. S. Diercks , S. Wuttke , Angew. Chem., Int. Ed. Engl. 2021, 60, 23975; 33989445 10.1002/anie.202106259

[37] S. Sikiru , T. L. Oladosu , T. I. Amosa , J. O. Olutoki , M. N. M. Ansari , K. J. Abioye , Z. U. Rehman , H. Soleimani , Int. J. Hydrogen Energy 2024, 56, 1152.

[38] H. Wang , X. Zhang , W. Zhang , M. Zhou , H. L. Jiang , Angew. Chem., Int. Ed. Engl. 2024, 63, e202401443. 38407530 10.1002/anie.202401443

[39] A. J. N. Esswein , D. G. Nocera , Chem. Rev. 2007, 107, 4022. 17927155 10.1021/cr050193e

[40] a) A. Dhakshinamoorthy , Z. Li , S. Yang , H. Garcia , Chem. Soc. Rev. 2024, 53, 3002; 38353930 10.1039/d3cs00205e

[41] Y.‐C. He , J. Zhang , L.‐Y. Xiao , Z.‐H. Yuan , Y. Yu , Y. Wang , K. Zhu , W.‐Q. Kan , CrystEngComm 2019, 21, 7166.

[42] H. Lu , G. Peng , J. Zou , L. Cao , Y. Xie , L. Zhang , S. You , F. Gao , Inorg. Chem. 2024, 63, 16243. 39159300 10.1021/acs.inorgchem.4c02118

[43] X. Qiu , J. Xie , X. Ning , Y. Cao , Z. Lu , J. Hu , A. Hao , Appl. Surf. Sci. 2024, 670, 160694.

[44] A. J. Rieth , K. M. Hunter , M. Dinca , F. Paesani , Nat. Commun. 2019, 10, 4771. 31628319 10.1038/s41467-019-12751-z PMC6802106

[45] L. Gudiño , M. Peñas‐Garzón , J. J. Rodriguez , J. Bedia , C. Belver , Catal. Commun. 2024, 187, 106858.

[46] J.‐H. Wang , F. Kong , B.‐F. Liu , S.‐N. Zhuo , N.‐Q. Ren , H.‐Y. Ren , Environ. Sci.: Nano 2024, 11, 3286.

[47] X. Yu Gao , Y. Wang , E. Wu , C. Wang , B. Li , Y. Zhou , B. Chen , P. Li , Angew. Chem., Int. Ed. Engl. 2023, 62, e202312393. 37773007 10.1002/anie.202312393

[48] Q. Guan , L. L. Zhou , Y. B. Dong , Chem. Soc. Rev. 2022, 51, 6307. 35766373 10.1039/d1cs00983d

[49] R. N. Yadav , A. K. Srivastava , S. Dey , A. Das , B. K. Banik , M. F. Hossain , Coord. Chem. Rev. 2024, 520, 216136.

[50] P. W. Seo , I. Ahmed , S. H. Jhung , Chem. Eng. J. 2016, 299, 236.

[51] S. Hu , M. Liu , X. Guo , K. Li , Y. Han , C. Song , G. Zhang , Cryst. Growth Des. 2017, 17, 6586.

[52] G. Zhan , L. Fan , F. Zhao , Z. Huang , B. Chen , X. Yang , S. F. Zhou , Adv. Funct. Mater. 2019, 29, 1806720.

[53] J. B. Decoste , G. W. Peterson , M. W. Smith , C. A. Stone , C. R. Willis , J. Am. Chem. Soc. 2012, 134, 1486. 22239201 10.1021/ja211182m

[54] Q. Xiong , Y. Chen , D. Yang , K. Wang , Y. Wang , J. Yang , L. Li , J. Li , Mater. Chem. Front. 2022, 6, 2944.

[55] J. Li , S. Cheng , Q. Zhao , P. Long , J. Dong , Int. J. Hydrogen Energy 2009, 34, 1377.

[56] Q. Sun , L. Qin , C. Lai , S. Liu , W. Chen , F. Xu , D. Ma , Y. Li , S. Qian , Z. Chen , W. Chen , H. Ye , J. Hazard. Mater. 2023, 447, 130848. 36696779 10.1016/j.jhazmat.2023.130848

[57] I. Ahmed , S. H. Jhung , Chem. Eng. J. 2017, 310, 197.

[58] Z. J. Lin , J. Lu , M. Hong , R. Cao , Chem. Soc. Rev. 2014, 43, 5867. 24699533 10.1039/c3cs60483g

[59] Q. Qiu , T. Wang , L. Jing , K. Huang , D. Qin , Int. J. Hydrogen Energy 2020, 45, 11077.

[60] L. Figueroa‐Quintero , D. Villalgordo‐Hernandez , J. J. Delgado‐Marin , J. Narciso , V. K. Velisoju , P. Castano , J. Gascon , E. V. Ramos‐Fernandez , Small Methods 2023, 7, e2201413. 36789569 10.1002/smtd.202201413

[61] Y. Shen , T. Pan , L. Wang , Z. Ren , W. Zhang , F. Huo , Adv. Mater. 2021, 33, 2007442. 10.1002/adma.202007442 34050572

[62] X.‐W. Zhu , D. Luo , X.‐P. Zhou , D. Li , Coord. Chem. Rev. 2022, 455, 214354.

[63] S. Daliran , A. R. Oveisi , Y. Peng , A. Lopez‐Magano , M. Khajeh , R. Mas‐Balleste , J. Aleman , R. Luque , H. Garcia , Chem. Soc. Rev. 2022, 51, 8140. 36004669 10.1039/d2cs90071h

[64] I. V. Sterkhova , L. N. Parshina , L. A. Grishchenko , T. N. Borodina , L. A. Belovezhets , V. A. Semenov , Struct. Chem. 2023, 34, 2249.

[65] Y.‐R. Lee , M.‐S. Jang , H.‐Y. Cho , H.‐J. Kwon , S. Kim , W.‐S. Ahn , Chem. Eng. J. 2015, 271, 276.

[66] R. N. Ali , W. A. Qureshi , M. Yaseen , H. Jiang , L. Wang , J. Yang , Q. Liu , Mater. Today Sustainability 2023, 22, 100337.

[67] A. Ruduss , A. Jece , K. A. Stucere , K.‐W. Chen , B. Turovska , S. Belyakov , A. Vembris , C.‐H. Chang , K. Traskovskis , J. Mater. Chem. C 2024, 12, 2968.

[68] M. Huang , Y. Yang , H. Cao , M. Yuan , T. Ye , L. Hao , J. Yu , F. Yin , F. Xu , X. Wu , J. Environ. Chem. Eng. 2024, 12, 113750.

[69] M. Koy , P. Bellotti , M. Das , F. Glorius , Nat. Catal. 2021, 4, 352.

[70] M.‐H. Yu , X.‐T. Liu , B. Space , Z. Chang , X.‐H. But , Coord. Chem. Rev. 2021, 427, 213518.

[71] G. Sathiyan , G. Venkatesan , S. K. Ramasamy , J. Lee , S. Bharathi , J. Environ. Chem. Eng. 2024, 12, 112804.

[72] T. Boruah , S. K. Das , G. Kumar , S. Mondal , R. S. Dey , Chem. Commun. (Cambridge, U. K.) 2022, 58, 5506. 10.1039/d2cc00865c 35419579

[73] L. Yue , X. Wang , L. Chen , D. Shen , Z. Shao , H. Wu , S. Xiao , W. Liang , Y. Yu , Y. Li , Energy Environ. Sci. 2024, 17, 1117.

[74] H. G. Jin , P. C. Zhao , Y. Qian , J. D. Xiao , Z. S. Chao , H. L. Jiang , Chem. Soc. Rev. 2024, 53, 9378. 39163028 10.1039/d4cs00095a

[75] M. I. Anwar , M. Asad , L. Ma , W. Zhang , A. Abbas , M. Y. Khan , M. Zeeshan , A. Khatoon , R. Gao , S. Manzoor , M. Naeem Ashiq , S. Hussain , M. Shahid , G. Yang , Coord. Chem. Rev. 2023, 478, 214967.

[76] X. L. Xu , N. N. Wang , Y. H. Zou , X. Qin , P. Wang , X. Y. Lu , X. Y. Zhang , W. Y. Sun , Y. Lu , Nat. Commun. 2024, 15, 7273. 39179619 10.1038/s41467-024-51552-x PMC11344049

[77] T. Li , Z. Jin , J. Colloid Interface Sci. 2022, 605, 385. 34332412 10.1016/j.jcis.2021.07.098

[78] W. A. Qureshi , R. N. Ali , S. N.‐U.‐Z. Haider , N. Ahmad , M. U. Khan , L. Wang , C. Cheng , S. Rao , A. Ali , Q. Q. Liu , J. Yang , Mater. Today Sustainability 2024, 25, 100679.

[79] E. N. Musa , A. K. Yadav , K. T. Smith , M. S. Jung , W. F. Stickle , P. Eschbach , X. Ji , K. Stylianou , Angew. Chem., Int. Ed. Engl. 2024, 63, e202405681. 38985847 10.1002/anie.202405681

[80] X. Hou , J. Wang , B. Mousavi , N. Klomkliang , S. Chaemchuen , Dalton Trans. 2022, 51, 8133. 35551351 10.1039/d2dt01030e

[81] Y. Xin , Y. Cao , J. Yang , X. Guo , K. Shen , W. Yao , J. Mater. Chem. A 2024, 12, 4931.

[82] J. Yi , G. Lee , S. S. Park , Small Methods 2024, 8, 2400363. 10.1002/smtd.202400363 38803311

[83] M. Wen , N. Sun , L. Jiao , S. Q. Zang , H. L. Jiang , Angew. Chem., Int. Ed. Engl. 2024, 63, e202318338. 38230982 10.1002/anie.202318338

[84] Y. Liu , Y. Wei , M. Liu , Y. Bai , X. Wang , S. Shang , J. Chen , Y. Liu , Angew. Chem., Int. Ed. Engl. 2021, 60, 2887. 33300656 10.1002/anie.202012971

[85] S. Głowniak , B. Szczęśniak , J. Choma , M. Jaroniec , Mater. Today 2021, 46, 109. 10.1002/adma.202103477 34580939

[86] H. Su , J. Hou , J. Zhu , Y. Zhang , B. Van der Bruggen , Sep. Purif. Technol. 2024, 333, 125957.

[87] B. Singh , H. Gupta , Chem. Commun. (Cambridge, U. K.) 2024, 60, 8020. 10.1039/d4cc02729a 38994743

[88] L. Mao , J. Qian , Small 2024, 20, 2308732. 10.1002/smll.202308732 38072778

[89] R. Kumar , R. Singh , S. Dutta , Energy Fuels 2024, 38, 2601.

[90] Y. An , X. Lv , W. Jiang , L. Wang , Y. Shi , X. Hang , H. Pang , Green Chem. Eng. 2024, 5, 187.

[91] L. Sun , X. Pan , Y. Xie , J. Zheng , S. Xu , L. Li , G. Zhao , Angew. Chem., Int. Ed. 2024, 63, e202402176. 10.1002/anie.202402176 38470010

[92] P. A. Krisbiantoro , T.‐J. Kuo , Y.‐C. Chang , W. Liao , J.‐P. Sun , C.‐Y. Yang , Y. Kamiya , F.‐K. Shieh , C.‐C. Chen , K. C. W. Wu , Mater. Today Nano 2024, 25, 100459.

[93] C. A. Anyama , H. Louis , B. E. Inah , T. E. Gber , J. O. Ogar , A. A. Ayi , J. Mol. Struct. 2023, 1277, 134825.

[94] L.‐Z. Wu , X.‐Y. Zhou , P.‐C. Zeng , J.‐Y. Huang , M.‐D. Zhang , L. Qin , Polyhedron 2022, 225, 116035.

[95] Q. Qi , Z. Liu , X. Chen , J. Yu , X. Li , R. Wang , Y. Liu , J. Chen , Biosens. Bioelectron. 2024, 264, 116693. 39167887 10.1016/j.bios.2024.116693

[96] Y. Y. Tang , X. Luo , R. Q. Xia , J. Luo , S. K. Peng , Z. N. Liu , Q. Gao , M. Xie , R. J. Wei , G. H. Ning , D. Li , Angew. Chem., Int. Ed. Engl. 2024, 63, e202408186. 38895811 10.1002/anie.202408186

[97] R. Ye , L. Ma , J. Mao , X. Wang , X. Hong , A. Gallo , Y. Ma , W. Luo , B. Wang , R. Zhang , M. S. Duyar , Z. Jiang , J. Liu , Nat. Commun. 2024, 15, 2159. 38461315 10.1038/s41467-024-46513-3 PMC10924954

[98] N. Rosi , Y. He , M. De Souza , T. Luo , S. Achar , J. K. Johnson , Angew. Chem., Int. Ed. Engl. 2024, 63, e202409150. 39046732 10.1002/anie.202409150

[99] Z. Qiu , Y. Li , Y. Gao , Z. Meng , Y. Sun , Y. Bai , N. T. Suen , H. C. Chen , Y. Pi , H. Pang , Angew. Chem., Int. Ed. Engl. 2023, 62, e202306881. 37389975 10.1002/anie.202306881

[100] J. Xing , L. Schweighauser , S. Okada , K. Harano , E. Nakamura , Nat. Commun. 2019, 10, 3608. 31444338 10.1038/s41467-019-11564-4 PMC6707309

[101] T. Zelenka , M. Balaz , M. Ferova , P. Diko , J. Bednarcik , A. Kiralyova , L. Zauska , R. Bures , P. Sharda , N. Kiraly , A. Badac , J. Vyhlidalova , M. Zelinska , M. Almasi , Sci. Rep. 2024, 14, 15386. 38965298 10.1038/s41598-024-66432-z PMC11224341

[102] C. Yang , S. Shang , L. Lin , P. Wang , Z. Ye , Y. Wang , K. Shih , L. Sun , X. Li , Nat. Water 2024, 2, 793.

[103] W. Cheng , H. Zhang , D. Luan , X. W. Lou , Sci. Adv. 2021, 7, 2580. 10.1126/sciadv.abg2580 PMC8081372 33910899

[104] Z. Xia , B. Shi , W. Zhu , Y. Xiao , C. Lü , Adv. Funct. Mater. 2022, 32, 2207655.

[105] a) M. Yahia , L. A. Lozano , J. M. Zamaro , C. Téllez , J. Coronas , Sep. Purif. Technol. 2024, 330, 125558;

[106] W.‐T. Li , C.‐X. Wu , Y.‐J. Zhang , H. Guo , Z. Zhao , M.‐L. Chen , Microchem. J. 2023, 191, 108925.

[107] M. M. Mahmoud , Next Mater. 2025, 6, 100316.

[108] D. Yu , C. Duan , B. Gu , Renewable Energy 2023, 219, 119338.

[109] W. T. Jung , Y. H. Hsieh , Y. J. Kuo , Y. H. Yu , Y. H. Liu , K. L. Lu , H. L. Lee , Talanta 2023, 263, 124733. 37247453 10.1016/j.talanta.2023.124733

[110] Y. Li , Y. Zhang , Z. Wang , C. Zhang , F. Meng , J. Zhao , X. Li , J. Hu , Appl. Catal., A 2024, 683, 119851.

[111] Z. Liu , J. Wang , S. Dong , L. Wang , L. Li , Z. Cao , Y. Zhang , L. Cheng , J. Yang , Ultrason. Sonochem. 2024, 107, 106912. 38762940 10.1016/j.ultsonch.2024.106912 PMC11130732

[112] C. Xie , H. Li , B. Niu , H. Guo , X. Lin , J. Alloys Compd. 2024, 989, 174363.

[113] M. Ishfaq , S. A. Khan , M. A. Nazir , S. Ali , M. Younas , M. Mansha , S. S. A. Shah , M. Arshad , A. Rehman , J. Mol. Struct. 2024, 1301, 137384.

[114] Y. Ogura , K. Taniya , T. Horie , K. L. Tung , S. Nishiyama , Y. Komoda , N. Ohmura , Ultrason. Sonochem. 2023, 96, 106443. 37216790 10.1016/j.ultsonch.2023.106443 PMC10213375

[115] Q. Wang , Y. Sun , S. Li , P. Zhang , Q. Yao , RSC Adv. 2020, 10, 37600. 35515141 10.1039/d0ra07950b PMC9057214

[116] G. Li , G. Han , L. Wang , X. Cui , N. K. Moehring , P. R. Kidambi , D. E. Jiang , Y. Sun , Nat. Commun. 2023, 14, 525. 36720867 10.1038/s41467-023-36142-7 PMC9889775

[117] C. Li , H. Zhang , M. Liu , F.‐F. Lang , J. Pang , X.‐H. But , Ind. Chem. Mater. 2023, 1, 9.

[118] Y. Li , Y. Wu , T. Li , M. Lu , Y. Chen , Y. Cui , J. Gao , G. Qian , Carbon Energy 2022, 5, e265.

[119] J. Z. X. Heng , T. T. Y. Tan , X. Li , W. W. Loh , Y. Chen , Z. Xing , Z. Lim , J. L. Y. Ong , K. S. Lin , Y. Nishiyama , T. Yoshida , L. Zhang , K. I. Otake , S. Kitagawa , X. J. Loh , E. Ye , J. Y. C. Lim , Angew. Chem., Int. Ed. Engl. 2024, 63, e202408718. 39088314 10.1002/anie.202408718

[120] W. Zheng , L. Y. S. Lee , ACS Energy Lett. 2021, 6, 2838.

[121] a) Y. Guo , Q. Huang , J. Ding , L. Zhong , T. T. Li , Y. Hu , J. Qian , S. Huang , Int. J. Hydrogen Energy 2021, 46, 2182;

[122] C. Han , X. Zhu , J. Ding , T. Miao , S. Huang , J. Qian , Inorg. Chem. 2022, 61, 18350. 36350270 10.1021/acs.inorgchem.2c03651

[123] P. Sharma , M. Sharma , M. Dearg , M. Wilding , T. J. A. Slater , C. R. A. Catlow , Angew. Chem., Int. Ed. Engl. 2023, 62, e202301239. 36788107 10.1002/anie.202301239

[124] a) J. Guo , Q. Xia , W. Y. Tang , Z. Li , X. Wu , L.‐J. Liu , W.‐P. To , H.‐X. Shu , K.‐H. Low , P. C. Y. Chow , T. W. B. Lo , J. He , Nat. Catal. 2024, 7, 307;

[125] T. He , W. Zhen , Y. Chen , Y. Guo , Z. Li , N. Huang , Z. Li , R. Liu , Y. Liu , X. Lian , C. Xue , T. C. Sum , W. Chen , D. Jiang , Nat. Commun. 2023, 14, 329. 36658157 10.1038/s41467-023-35999-y PMC9852592

[126] S. Guo , J. Zhang , G. Fan , A. Shen , X. Wang , Y. Guo , J. Ding , C. Han , X. Gu , L. Wu , Angew. Chem., Int. Ed. Engl. 2024, 63, e202409698. 38924667 10.1002/anie.202409698

[127] E. Morais , C. O’Modhrain , K. R. Thampi , J. A. Sullivan , J. Catal. 2021, 401, 288.

[128] P. Lu , K. Liu , Y. Liu , Z. Ji , X. Wang , B. Hui , Y. Zhu , D. Yang , L. Jiang , Appl. Catal., B 2024, 63, 345.

[129] M. A. A. El‐Khair , A. G. Al‐Gamal , K. I. Kabel , W. S. Gado , A. S. Morshedy , Int. J. Hydrogen Energy 2025, 101, 280.

[130] X. C. Cao , B. C. Zhang , J. Cui , C. Suo , X. C. Duan , S. H. Guo , X. M. Zhang , Langmuir 2024, 40, 18695. 39172768 10.1021/acs.langmuir.4c02335

[131] S. Qi , K. Zhu , T. Xu , H. Zhang , X. Guo , J. Wang , F. Zhang , X. Zong , Adv. Mater. 2024, 36, 2403328. 10.1002/adma.202403328 38586929

[132] a) P. Rassu , X. Ma , B. Wang , Coord. Chem. Rev. 2022, 465, 214561;

[133] a) H. H. Keypour , R. Karimi‐Nami , J. Kouhdareh , S. Bbaei , A. Maryamabadi , S. Alavinia , M. T. Rezaei , S. Shokri , Chem Pap. 2023, 77, 12;

[134] T. N. Tu , M. Scheer , Chem 2023, 9, 227.

[135] a) J.‐Y. Xian , X.‐X. Xie , Z.‐Y. Huang , Y.‐L. Liu , H.‐Y. Song , Z.‐Q. Chen , Y.‐C. Ou , S.‐R. Zheng , Cryst. Growth Des. 2023, 23, 1448;

[136] H. B. Luo , Q. Ren , P. Wang , J. Zhang , L. Wang , X. M. Ren , ACS Appl. Mater. Interfaces 2019, 11, 9164. 30747511 10.1021/acsami.9b01075

[137] A. Phan , C. J. Doonan , F. J. Uribe‐Romo , C. B. Knobler , M. O’Keeffe , O. M. Yaghi , Acc. Chem. Res. 2009, 43, 58. 10.1021/ar900116g 19877580

[138] H. Jiang , J. Jia , A. Shkurenko , Z. Chen , K. Adil , Y. Belmabkhout , L. J. Weselinski , A. H. Assen , D. X. Xue , M. O’Keeffe , M. Eddaoudi , J. Am. Chem. Soc. 2018, 140, 8858. 29923711 10.1021/jacs.8b04745

[139] B. Wang , X. L. Lv , D. Feng , L. H. Xie , J. Zhang , M. Li , Y. Xie , J. R. Li , H. C. Zhou , J. Am. Chem. Soc. 2016, 138, 6204. 27090616 10.1021/jacs.6b01663

[140] H. Liu , C. Xu , D. Li , H. L. Jiang , Angew. Chem., Int. Ed. Engl. 2018, 57, 5379. 29508919 10.1002/anie.201800320

[141] G. A. McCarver , M. J. Kramer , T. Yildirim , W. Zhou , Chem. Mater. 2024, 36, 8098. 10.1021/acs.chemmater.4c01699 PMC13344112 42421745

[142] S. Mahajan , M. Lahtinen , Cryst. Growth Des. 2023, 24, 747.

[143] E. E. Moushi , A. Kourtellaris , E. Andreou , A. Fidelli , G. S. Papaefstathiou , J. C. Plakatouras , A. J. Tasiopoulos , CrystEngComm 2020, 22, 2083.

[144] B. Zhang , S. Song , W. Li , L. Zheng , X. Ma , Ionics 2021, 27, 3553.

[145] W. Li , X. Guo , P. Geng , M. Du , Q. Jing , X. Chen , G. Zhang , H. Li , Q. Xu , P. Braunstein , H. Pang , Adv. Mater. 2021, 33, 2105163. 10.1002/adma.202105163 34554610

[146] W. J. Wang , Z. H. Sun , S. C. Chen , J. F. Qian , M. Y. He , Q. Chen , Appl. Organomet. Chem. 2021, 35, e6288.

[147] S. Ko , F. Gao , X. Yao , H. Yi , X. Tang , C. Wang , H. Liu , N. Luo , Z. Qi , New J. Chem. 2022, 46, 15758.

[148] Q. Li , Y. Liu , S. Niu , C. Li , C. Chen , Q. Liu , J. Huo , J. Colloid Interface Sci. 2021, 603, 148. 34186392 10.1016/j.jcis.2021.06.102

[149] L. Zhao , S. Liu , L. Wei , H. He , B. Jiang , Z. Zhan , J. Wang , X. Li , W. Gou , Catal. Lett. 2024, 154, 5294.

[150] J. Hu , H.‐X. Lao , X.‐W. Xu , W.‐K. Wang , L.‐L. Wang , Q.‐Q. Liu , Rare Met. 2024, 43, 2682.

[151] S. Dong , X. Liu , X. Kong , F. Dong , Y. Yu , L. Wang , D. Wang , Z. He , S. Song , Environ. Sci. Pollut. Res. Int. 2023, 30, 111039. 37801244 10.1007/s11356-023-30258-5

[152] C. M. Rueda‐Navarro , M. Cabrero‐Antonino , P. Escamilla , V. Díez‐Cabanes , D. Fan , P. Atienzar , B. Ferrer , I. Vayá , G. Maurin , H. G. Baldoví , S. Navalón , Nano Res. 2023, 17, 4134.

